# Biallelic *PAX5* mutations cause hypogammaglobulinemia, sensorimotor deficits, and autism spectrum disorder

**DOI:** 10.1084/jem.20220498

**Published:** 2022-08-10

**Authors:** Fabian M.P. Kaiser, Sarah Gruenbacher, Maria Roa Oyaga, Enzo Nio, Markus Jaritz, Qiong Sun, Wietske van der Zwaag, Emanuel Kreidl, Lydia M. Zopf, Virgil A.S.H. Dalm, Johan Pel, Carolin Gaiser, Rick van der Vliet, Lucas Wahl, André Rietman, Louisa Hill, Ines Leca, Gertjan Driessen, Charlie Laffeber, Alice Brooks, Peter D. Katsikis, Joyce H.G. Lebbink, Kikuë Tachibana, Mirjam van der Burg, Chris I. De Zeeuw, Aleksandra Badura, Meinrad Busslinger

**Affiliations:** 1 Department of Immunology, Erasmus MC, Rotterdam, Netherlands; 2 Research Institute of Molecular Pathology, Vienna BioCenter, Vienna, Austria; 3 Department of Neuroscience, Erasmus MC, Rotterdam, Netherlands; 4 Vienna BioCenter PhD Program, Doctoral School of the University of Vienna and Medical University of Vienna, Vienna, Austria; 5 Spinoza Centre for Neuroimaging, Amsterdam, Netherlands; 6 Vienna BioCenter Core Facilities, Vienna BioCenter, Vienna, Austria; 7 Division of Allergy and Clinical Immunology, Department of Internal Medicine, Erasmus MC, Rotterdam, Netherlands; 8 Department of Child and Adolescent Psychiatry, Erasmus MC, Rotterdam, Netherlands; 9 Department of Clinical Genetics, Erasmus MC, Rotterdam, Netherlands; 10 Department of Neurology, Erasmus MC, Rotterdam, Netherlands; 11 Department of Pediatrics, Erasmus MC, Rotterdam, Netherlands; 12 Department of Pediatrics, Maastricht University Medical Center, Maastricht, Netherlands; 13 Department of Molecular Genetics, Oncode Institute, Cancer Institute, Erasmus MC, Rotterdam, Netherlands; 14 Department of Radiation Oncology, Erasmus MC, Rotterdam, Netherlands; 15 Institute of Molecular Biotechnology of the Austrian Academy of Sciences, Vienna BioCenter, Vienna, Austria; 16 Department of Pediatrics, Leiden University Medical Center, Leiden, Netherlands; 17 Netherlands Institute for Neuroscience, Amsterdam, Netherlands

## Abstract

The genetic causes of primary antibody deficiencies and autism spectrum disorder (ASD) are largely unknown. Here, we report a patient with hypogammaglobulinemia and ASD who carries biallelic mutations in the transcription factor PAX5. A patient-specific *Pax5* mutant mouse revealed an early B cell developmental block and impaired immune responses as the cause of hypogammaglobulinemia. *Pax5* mutant mice displayed behavioral deficits in all ASD domains. The patient and the mouse model showed aberrant cerebellar foliation and severely impaired sensorimotor learning. PAX5 deficiency also caused profound hypoplasia of the substantia nigra and ventral tegmental area due to loss of GABAergic neurons, thus affecting two midbrain hubs, controlling motor function and reward processing, respectively. Heterozygous *Pax5* mutant mice exhibited similar anatomic and behavioral abnormalities. Lineage tracing identified Pax5 as a crucial regulator of cerebellar morphogenesis and midbrain GABAergic neurogenesis. These findings reveal new roles of Pax5 in brain development and unravel the underlying mechanism of a novel immunological and neurodevelopmental syndrome.

## Teaser

A mouse model carrying biallelic *Pax5* mutations identified in a patient with hypogammaglobulinemia and autism spectrum disorder shows a B cell developmental arrest and autistic-like behavior caused by abnormal development of the cerebellum and loss of ventral midbrain GABAergic neurons.

## Graphical Abstract

**Figure:**
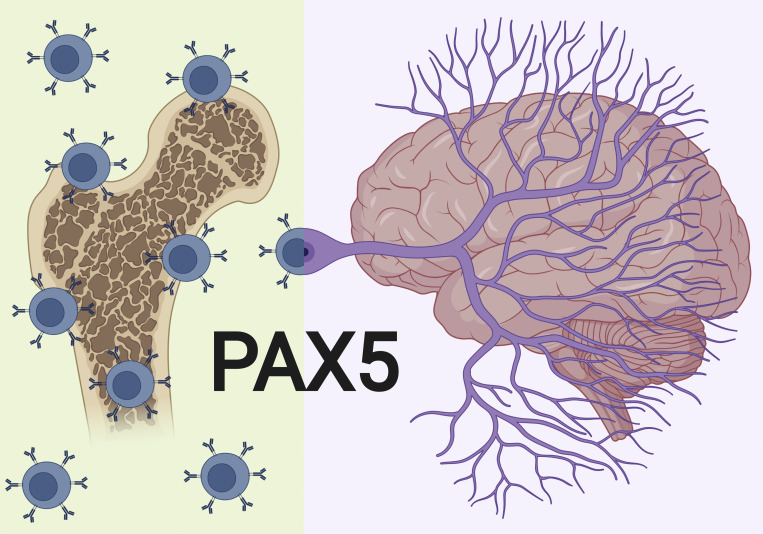

## Introduction

Autism spectrum disorder (ASD) refers to a heterogeneous continuum of neurodevelopmental abnormalities characterized by social, cognitive, and behavioral features, which include impaired communication skills, abnormal social interactions, and repetitive and stereotyped actions (Quesnel-Vallieres et al., 2019; Vorstman et al., 2017; Wang et al., 2014). The etiology of ASD has a strong genetic component, as ∼5% of ASD individuals carry de novo or inherited mutations in known ASD-causing loci, and single nucleotide or copy number variants in candidate ASD risk genes have been found in ∼25% of all ASD cases (Quesnel-Vallieres et al., 2019; Vorstman et al., 2017). Based on phenotypic heterogeneity and genetic complexity, ASD is considered to be primarily a multifactorial disorder. *PAX5* has been identified as a candidate ASD risk gene by the discovery of heterozygous *PAX5* mutations in individuals with ASD (Gofin et al., 2022; Iossifov et al., 2012; O’Roak et al., 2014; Stessman et al., 2017). Here, we demonstrate that *PAX5* mutations can cause a monogenic form of ASD.

During embryogenesis, the transcription factor Pax5 is expressed, together with the related Pax2 protein, in the isthmic organizer at the midbrain–hindbrain boundary (Urbánek et al., 1994) that controls the patterning and neuronal specification of the posterior midbrain and anterior hindbrain, from which the cerebellum develops (Zervas et al., 2005). *Pax5* mutant mice exhibit abnormal morphogenesis of the posterior midbrain and anterior cerebellum (Urbánek et al., 1994), while both the midbrain and cerebellum fail to develop in *Pax2*, *Pax5* double-mutant embryos due to lack of the isthmic organizer (Schwarz et al., 1997). Within the hematopoietic system, Pax5 is exclusively expressed in the B lymphoid lineage (Fuxa and Busslinger, 2007), where it functions as an essential regulator of B cell commitment (Nutt et al., 1999), development (Horcher et al., 2001), and immunity (Calderón et al., 2021). At the molecular level, Pax5 performs a dual role in B lymphopoiesis by acting as a transcriptional repressor to suppress B lineage–inappropriate genes (Delogu et al., 2006; Revilla-i-Domingo et al., 2012) and as an activator to induce gene expression required for B cell development and function (Revilla-i-Domingo et al., 2012; Schebesta et al., 2007). In mature B cells, Pax5 additionally promotes phosphoinositide 3-kinase (PI3K) signaling by down-regulating expression of the phosphatase and tensin homolog (PTEN) protein, a negative regulator of this pathway (Calderón et al., 2021). Another important function of Pax5 is to suppress B cell tumorigenesis in mice (Cobaleda et al., 2007) and humans (Mullighan et al., 2007), where heterozygous *PAX5* mutations prominently contribute to the development of B cell acute lymphoblastic leukemia (Gu et al., 2019). By identifying a patient with biallelic *PAX5* mutations, we now demonstrate that PAX5 deficiency can also cause neurodevelopmental abnormalities including ASD in addition to hypogammaglobulinemia.

## Results

### Characterization of a patient with biallelic *PAX5* mutations

**Figure 1.**
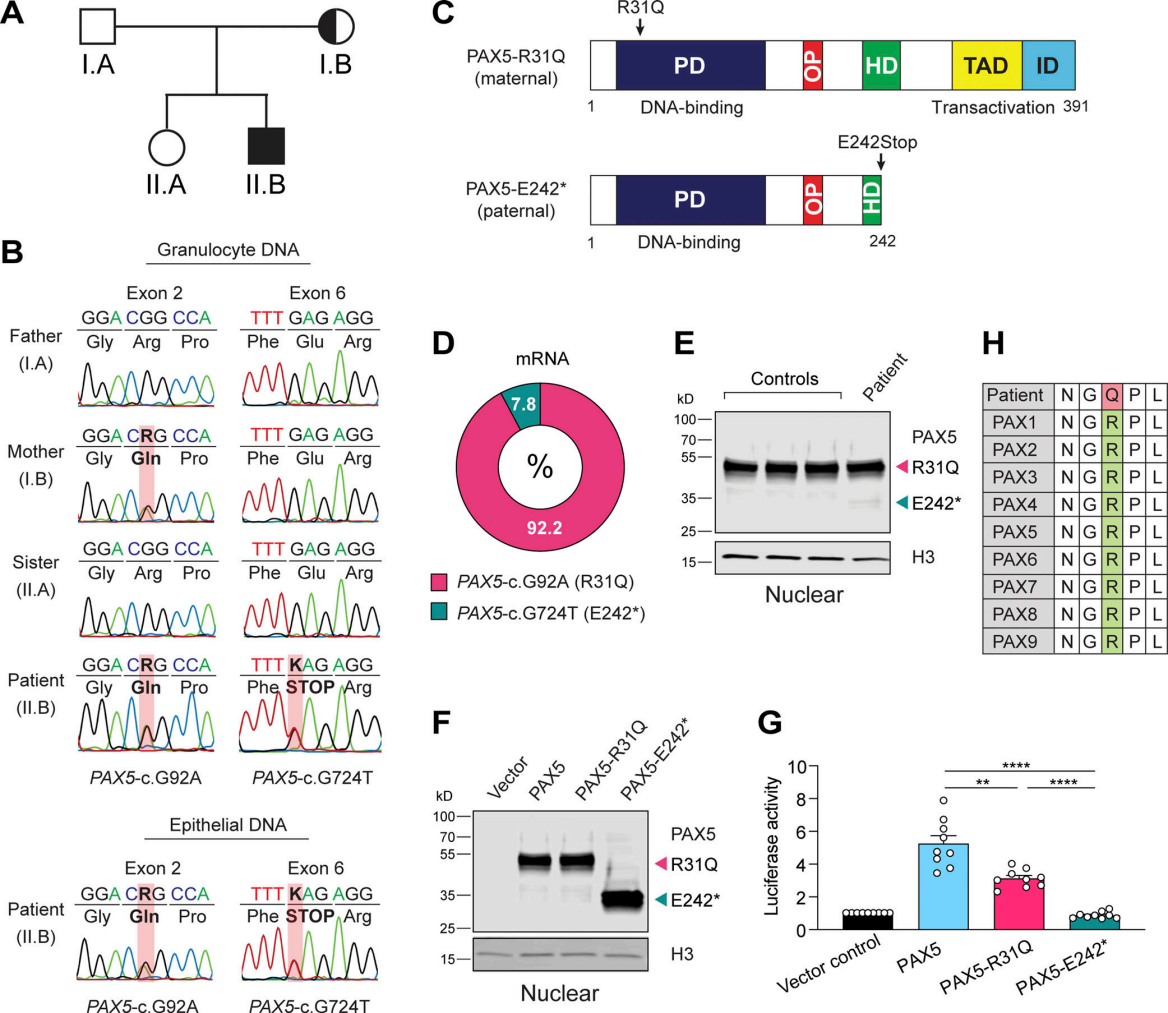
**Identification of a patient with biallelic *PAX5* mutations. (A)** Family pedigree of the 19-yr-old patient (II.B) with his asymptomatic mother (I.B). **(B)** Chromatograms of Sanger sequencing showing segregation of the *PAX5*-c.G92A (left) and *PAX5*-c.G724T (right) mutations in the four family members. PCR-amplified DNA from granulocytes was analyzed. In addition, PCR analysis was performed with DNA from epithelial cells of the patient (II.B). R (G + A); K (G + T). **(C)** PAX5 domain organization. The two mutations on the maternal and paternal *PAX5* alleles are indicated together with the paired domain (PD), octapeptide motif (OP), partial homeodomain (HD), transactivation domain (TAD), and inhibitory domain (ID; Dörfler and Busslinger, 1996). **(D)** Abundance of *PAX5*-c.G92A and *PAX5*-c.G724T mRNAs in naive mature B cells of the patient. The frequency of the mutant mRNAs was determined by RT-qPCR amplification and subsequent sequencing of the cloned PCR fragments. **(E)** Immunoblot analysis of nuclear extracts from EBV-immortalized B cells of the patient and three controls with anti-PAX5 and anti-H3 antibodies. The positions of the full-length PAX5-R31Q and truncated PAX5-E242* proteins are indicated. **(F)** Expression of wild-type PAX5, PAX5-R31Q, and PAX5-E242* proteins in nuclear extracts of transfected HEK-293T cells, analyzed by immunoblotting with anti-PAX5 and anti-H3 antibodies. **(G)** Analysis of the transactivation potential of the wild-type and mutant PAX5 proteins in HEK-293T cells, transfected with the indicated PAX5 expression vectors, the plasmid lucCD19 containing three high-affinity PAX5-binding sites upstream of the β-globin TATA box and initiator region linked to the firefly luciferase gene (Czerny and Busslinger, 1995), and a control renilla luciferase plasmid. The firefly and renilla luciferase activities were measured 2 d after transfection. Normalized firefly activity is shown relative to the pcDNA3.1 vector control (set to 1). The pooled data of three independent experiments (each dot corresponding to one transfection assay; *n* = 9 for each expression vector) are shown as mean values with SEM analyzed by ANOVA with Dunnett’s T3 multiple comparison test; **, P < 0.01; ****, P < 0.0001. For detailed statistical information, see Table S6. **(H)** Evolutionary conservation of arginine (R) at position 31 in all human PAX proteins; remaining amino acids are abbreviated as follows: asparagine (N), glycine (G), glutamine (Q), proline (P), leucine (L).

A male patient with recurrent infections at the age of 2.5 yr was diagnosed initially with hypogammaglobulinemia and later also with ASD, combined with sensorimotor and cognitive deficits (Fig. 1 A; patient description in Materials and methods; Tables S1 and S2). Whole-exome sequencing (WES) of peripheral blood mononuclear cells of the patient identified two mutations in the *PAX5* gene, which resulted in the missense mutation R31Q (*PAX*5-c.G92A) in the N-terminal part of the DNA-binding paired domain and in the nonsense mutation E242Stop (*PAX5*-c.G724T) in the partial homeodomain of PAX5 (Fig. 1, B and C). The asymptomatic mother (I.B) of the patient (II.B) carried the R31Q mutation (Fig. 1, A and B), while the de novo mutation E242Stop (referred to as E242*) was also detected in epithelial cells of the patient (Fig. 1 B), suggestive of its sporadic generation in the paternal germline. Quantitative RT-PCR (RT-qPCR) amplification and subsequent cloning of the entire PAX5-coding sequence from naive mature B cells of the patient’s blood revealed that the two mutations were present on separate *PAX5* alleles (Fig. 1 C). The same analysis furthermore demonstrated that the *PAX*5-c.G92A (R31Q) mRNA constituted 92.2% of all *PAX5* mRNA in naive mature B cells of the patient, as most of the *PAX5*-c.G724T (E242*) transcripts were apparently eliminated by nonsense-mediated mRNA decay induced by the presence of the premature stop codon (Fig. 1 D). Consequently, the PAX5 protein consisted almost exclusively of the PAX5-R31Q protein, as shown by immunoblot analysis of EBV-immortalized B cells of the patient (Fig. 1 E). The E242* protein could, however, be stably expressed in transfected HEK-293T cells (Fig. 1 F). In contrast to the PAX5-E242* protein lacking the C-terminal transactivation domain, the PAX5-R31Q protein was able to activate a PAX5-dependent luciferase reporter gene in transiently transfected HEK-293T cells, albeit less efficiently compared with the wild-type PAX5 protein (Fig. 1 G). The stringent conservation of the arginine (R) residue 31 among all nine PAX proteins (Fig. 1 H) suggested that it may critically contribute to the DNA-binding function of the paired domain. Together, these data demonstrate that the E242* substitution is likely a null mutation due to mRNA degradation and absent transactivation function, whereas the R31Q substitution may be a hypomorphic mutation leading to impaired DNA binding.

### B cell deficiency of the patient is caused by an early developmental arrest

**Figure 2.**
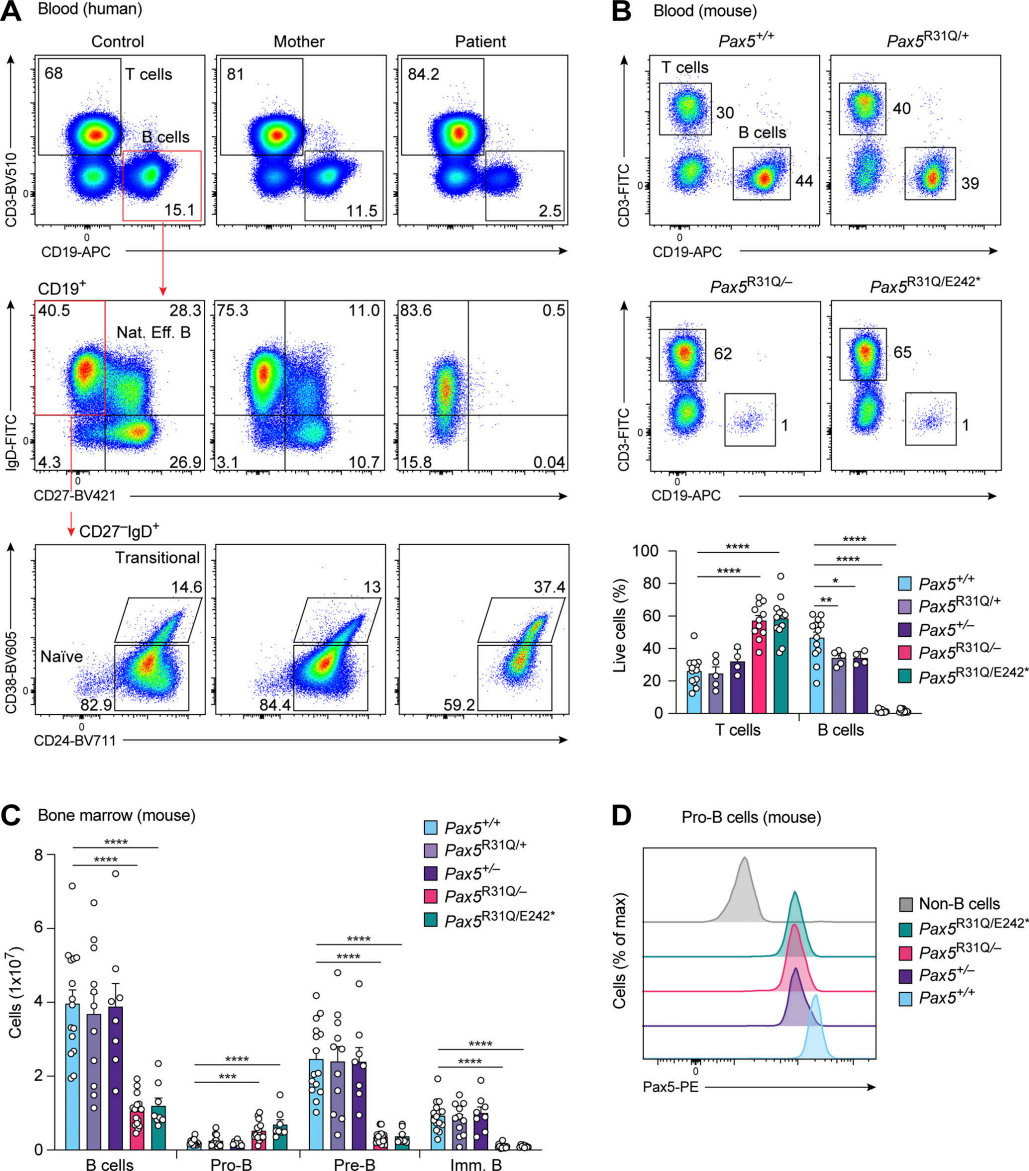
**Low numbers of B cells in the patient and early B cell development arrest in *Pax5***^R31Q/E242^* and *Pax5*^**R31Q/−**^ **mice. (A)** Analysis of peripheral blood from the patient, the mother, and a control. T (CD3^+^), B (CD19^+^), natural effector B (CD19^+^CD27^+^IgD^+^), transitional B (CD19^+^CD27^−^IgD^+^CD38^+^CD24^dim^), and naive mature B (CD19^+^CD27^−^IgD^+^CD38^−^CD24^−^) cells were identified by flow cytometry. Numbers refer to the percentage of cells in the indicated gate. One of three experiments is shown. **(B)** Flow-cytometric analysis of T (CD3^+^) and B (CD19^+^) cells in the blood from 8–10-wk-old mice of the indicated genotypes (upper panel). The frequencies of T and B cells are shown as mean values with SEM (lower panel; *n* ≥ 5 per genotype). **(C)** Flow-cytometric analysis of B cell development in the bone marrow of 3–4-wk-old mice of the indicated genotypes. Absolute numbers of total B, pro-B, pre-B, and immature (imm) B cells are shown as mean values with SEM (*n* ≥ 8 per genotype). Definitions of the different cell types are in Fig. S1 C and Materials and methods. **(D)** Analysis of Pax5 expression in pro-B cells of the indicated genotypes. Pax5 levels were determined by intracellular staining combined with flow-cytometric analysis. ANOVA with Tukey’s or Dunnett’s T3 multiple comparisons test (B and C); *, P < 0.05; **, P < 0.01; ***, P < 0.001; ****, P < 0.0001. For detailed statistical information, see Table S6. Each dot (B and C) corresponds to one mouse.

Consistent with the near-absence of serum immunoglobulins (see patient description in Materials and methods; Table S1), B cells were strongly reduced in the peripheral blood of the patient relative to the mother and a healthy control (Fig. 2 A). Detailed flow-cytometric analysis demonstrated that the patient’s B cells consisted of transitional and naive mature B cells that expressed lower levels of the B cell surface proteins CD19 and IgD compared with B cells of controls (Fig. 2 A). Antigen-experienced CD27^+^ B cells, including natural effector and memory B cells as well as plasmablasts, were absent in the blood of the patient (Fig. 2 A and Table S1).

**Figure S1.**
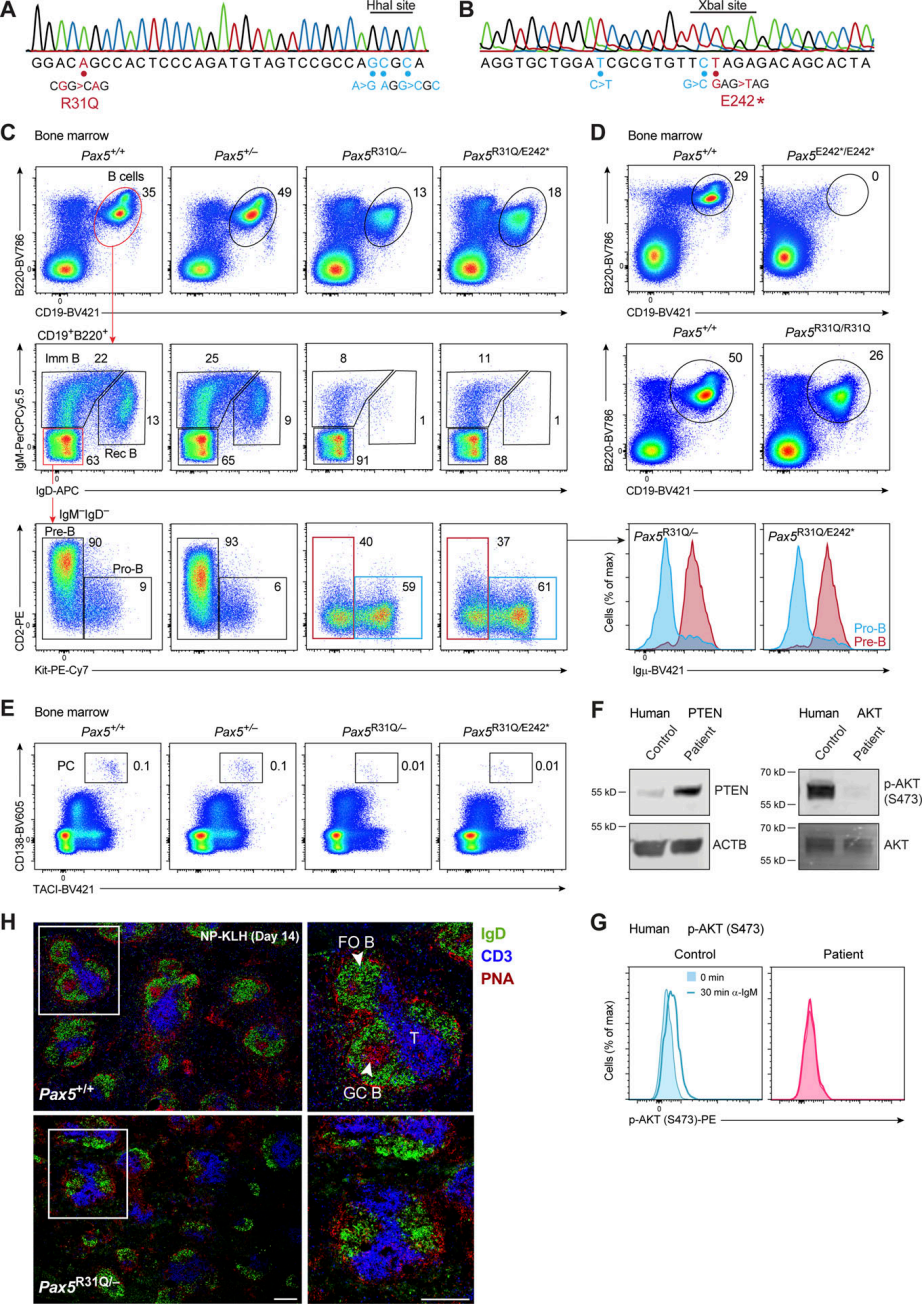
**B cell developmental arrest in *Pax5***^**R31Q/−**^ **and *Pax5***^R31Q/E242^* **mice. (A)** Generation of the *Pax5*^R31Q^ allele by introducing the R31Q mutation into the *Pax5* locus by CRISPR/Cas9-mediated mutagenesis in injected wild-type zygotes (see Materials and methods). The introduced mutation was verified by PCR amplification, cloning, and Sanger sequencing of genomic DNA from a *Pax5*^R31Q/+^ mouse. In addition to the R31Q mutation (red), three silent mutations (blue) were introduced to prevent Cas9 cleavage and to generate an HhaI restriction site (GCGC) for genotyping. **(B)** Generation of the *Pax5* ^E242^* by introducing the E242* mutation into the *Pax5* locus, as described in A. In addition to the E242* mutation (red), two silent mutations (blue) were introduced to prevent Cas9 cleavage and to generate an XbaI restriction site (TCTAGA) for genotyping. **(C)** Flow-cytometric analysis of the indicated B cell types in the bone marrow of 3–4-wk-old *Pax5*^+/+^, *Pax5*^+/−^, *Pax5*^R31Q/−^, and *Pax5*^R31Q/E242^* mice. Numbers refer to the percentage of cells in the indicated gate. Pre-B cells (B220^+^CD19^+^Kit^−^CD2^+^IgM^−^IgD^−^) are defined by the expression of CD2, which is, however, not expressed on *Pax5*^R31Q/−^ and *Pax5*^R31Q/E242^* pre-B cells, as *Cd2* is a directly activated Pax5 target gene (Revilla-i-Domingo et al., 2012). The B220^+^CD19^+^Kit^−^CD2^−^IgM^−^IgD^−^ B cells were identified as pre-B cells (red), as they expressed the in-frame rearranged Igμ protein in contrast to pro-B cells (blue), as shown to the right. Immature, imm; Rec, recirculating. **(D)** Loss of all B cells (B220^+^CD19^+^) in the bone marrow of *Pax5* ^E242^*^/E242^* mice in contrast to *Pax5*^R31Q/R31Q^ mice, as shown by flow cytometry. **(E)** Flow-cytometric analysis of long-lived plasma cells (PCs) in the bone marrow of nonimmunized mice of the indicated genotypes. The different B cell types were defined as shown in C and E and Materials and methods. **(F)** PTEN expression and AKT phosphorylation at Ser473 in EBV-immortalized B cells of the patient and a representative control, as revealed by immunoblot analysis of whole-cell extracts with the respective antibodies. The expression of β-actin (ACTB) and total AKT protein served as loading controls. One of two experiments is shown. Similar results were obtained with a second independently generated EBV-immortalized B cell culture of the patient and EBV-immortalized B cells of two additional controls. **(G)** Impaired PI3K-AKT signaling in B cells of the patient. B cells from the blood of the patient and a healthy control were left untreated (0 min) or stimulated for 30 min with anti-IgM before intracellular analysis of AKT phosphorylation at Ser473. One of two experiments is shown. **(H)** Immunohistological analysis of spleen sections from *Pax5*^+/+^ and *Pax5*^R31Q/−^ mice 14 d after NP-KLH immunization. The sections were stained with PNA (red) and antibodies detecting IgD (green) and CD3 (blue). A higher magnification of the region indicated by a white box (left) is shown to the right. FO B, GC B, and T cell zones are indicated. One of three experiments is shown. The scale bar represents 200 μm.

To gain insight into how the two human *PAX5* mutations affect B lymphopoiesis and brain development in the patient, we generated two corresponding mouse models in view of the fact that the human and mouse Pax5 proteins differ at three only amino acid positions (Adams et al., 1992). To this end, we created the *Pax5*^R31Q^ and *Pax5*^E242^* alleles by CRISPR/Cas9-mediated mutagenesis to generate the *Pax5*^R31Q/E242^* and *Pax5*^R31Q/−^ mouse models (Fig. S1, A and B; Materials and methods). The *Pax5*^R31Q/E242^* and *Pax5*^R31Q/−^ mice were viable and thriving in contrast to *Pax5*^−/−^ mice, which die at weaning age (Urbánek et al., 1994). Similar to the patient, the frequency of B cells in the peripheral blood of *Pax5*^R31Q/E242^* and *Pax5*^R31Q/−^ mice was strongly reduced compared with control *Pax5*^+/+^, *Pax5*^R31Q/+^, and *Pax5*^+/−^ mice, as shown by flow cytometry (Fig. 2 B). Moreover, total B cell numbers were 3.5-fold reduced in the bone marrow of *Pax5*^R31Q/E242^* and *Pax5*^R31Q/−^ mice relative to *Pax5*^+/+^, *Pax5*^R31Q/+^, and *Pax5*^+/−^ mice (Figs. 2 C and S1 C). Pro-B cells were, however, 3-fold increased, while pre-B and immature B cells were 7- and 9-fold decreased in *Pax5*^R31Q/E242^* and *Pax5*^R31Q/−^ mice compared with *Pax5*^+/+^, *Pax5*^R31Q/+^, and *Pax5*^+/−^ mice, respectively (Figs. 2 C and S1 C). Intracellular Pax5 staining demonstrated that the Pax5-R31Q protein in *Pax5*^R31Q/−^ pro-B cells was expressed at the same level as wild-type Pax5 in *Pax5*^+/−^ pro-B cells (Fig. 2 D). Notably, there was no further increase in Pax5 expression in *Pax5*^R31Q/E242^* pro-B cells relative to *Pax5*^R31Q/−^ pro-B cells, suggesting that the Pax5-E242* protein was also not expressed in mouse B cells (Fig. 2 D). In contrast to the *Pax5*^R31Q/R31Q^ mice, no B cells were generated in the bone marrow of *Pax5*^E242^*^/E242^* mice (Fig. S1 D), which were growth retarded and died at weaning age like *Pax5*^−/−^ mice (Urbánek et al., 1994). These data therefore provide conclusive evidence that *Pax5*^E242^* is a null allele and *Pax5*^R31Q^ is a hypomorphic allele. In summary, we conclude that B cell development was partially arrested at the transition from pro-B to pre-B cell in the *Pax5*^R31Q/E242^* and *Pax5*^R31Q/−^ mouse models.

### Strong reduction of mature B cell types in the two *Pax5* mutant mouse models

**Figure 3.**
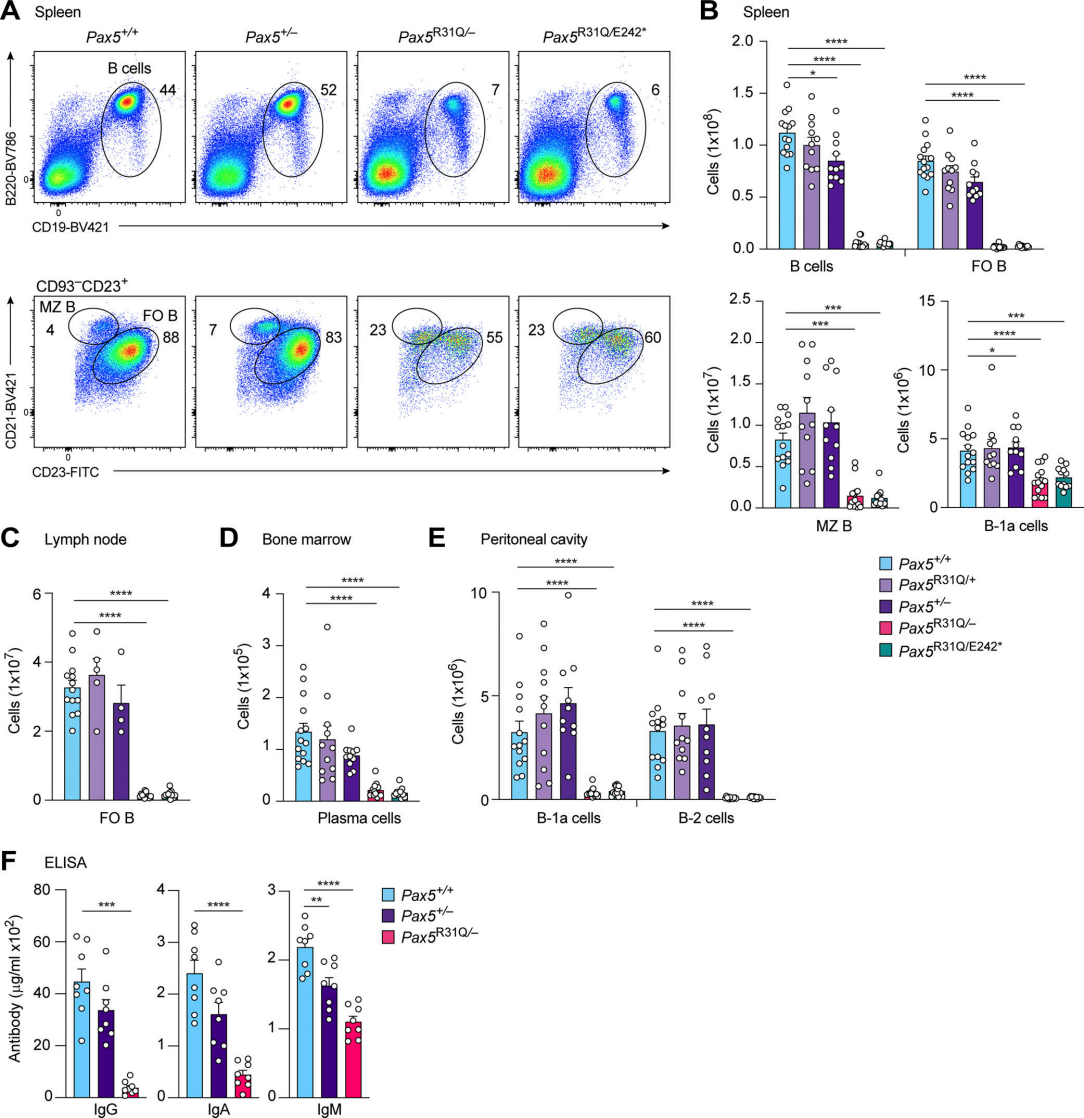
**Strong reduction of mature cell types in *Pax5***^R31Q/E242^* **and *Pax5***^**R31Q/−**^ **mice. (A)** Flow-cytometric analysis of total, MZ, and FO B cells in the spleen of nonimmunized *Pax5*^+/+^, *Pax5*^+/−^, *Pax5*^R31Q/−^, and *Pax5*^R31Q/E242^* mice. **(B–E)** Flow-cytometric analysis of the indicated B cell types in the spleen (B), lymph node (C), bone marrow (D), and peritoneal cavity (E) of nonimmunized mice of the indicated genotypes. Absolute cell numbers of total B, FO B, MZ B, B-1a, and conventional B-2 cells as well as plasma cells are shown as mean values with SEM (*n* ≥ 10 per genotype for B–E). The different B cell types were defined as shown in A, Fig. S1 E, and Materials and methods. **(F)** Titers of total IgG, IgA, and IgM antibodies in the serum of nonimmunized *Pax5*^+/+^, *Pax5*^+/−^, and *Pax5*^R31Q/−^ mice (8–10 wk). The antibody concentrations were determined by ELISA and are shown as mean values with SEM (*n* = 8 per genotype). ANOVA with Tukey’s, Dunnett’s or Dunnett’s T3 multiple comparisons test (B–F); *, P < 0.05; **, P < 0.01; ***, P < 0.001; ****, P < 0.0001. For detailed statistical information (B–F), see Table S6. Each dot (B–F) corresponds to one mouse.

Consistent with the low input of immature B cells from the bone marrow (Fig. 2 C), the numbers of total, follicular (FO), and marginal zone (MZ) B cells were greatly reduced in the spleen of nonimmunized *Pax5*^R31Q/E242^* and *Pax5*^R31Q/−^ mice compared with *Pax5*^+/+^, *Pax5*^R31Q/+^, and *Pax5*^+/−^ mice (Fig. 3, A and B). FO B cells in lymph nodes and long-lived plasma cells in the bone marrow were also strongly decreased in *Pax5*^R31Q/E242^* and *Pax5*^R31Q/−^ mice relative to control mice (Fig. 3, C and D; and Fig. S1 E). The innate-like B-1a cells of the mouse, for which no equivalent human B cell type has been conclusively identified, are responsible for the secretion of natural IgM antibodies, which function as a first line of defense by neutralizing pathogens (Baumgarth, 2011). In contrast to all other B cell types, B-1a cells were only twofold reduced in the spleen, but were 10-fold decreased in the peritoneal cavity of *Pax5*^R31Q/E242^* and *Pax5*^R31Q/−^ mice compared with control mice (Fig. 3, B and E). The minimal reduction of splenic B-1a cells is likely responsible for the minor decrease of the serum IgM level in nonimmunized *Pax5*^R31Q/−^ mice, whereas the serum IgG and IgA levels were strongly decreased in these mice relative to *Pax5*^+/+^ and *Pax5*^+/−^ mice (Fig. 3 F). In summary, all immunological data demonstrate that the *Pax5*^R31Q/E242^* and *Pax5*^R31Q/−^ mice are equivalent mouse models with regard to their B cell developmental defects in the bone marrow and peripheral lymphoid organs. We therefore performed all subsequent experiments with *Pax5*^R31Q/−^ mice.

### Absence of B cell immune responses in *Pax5*^R31Q/−^ mice

**Figure 4.**
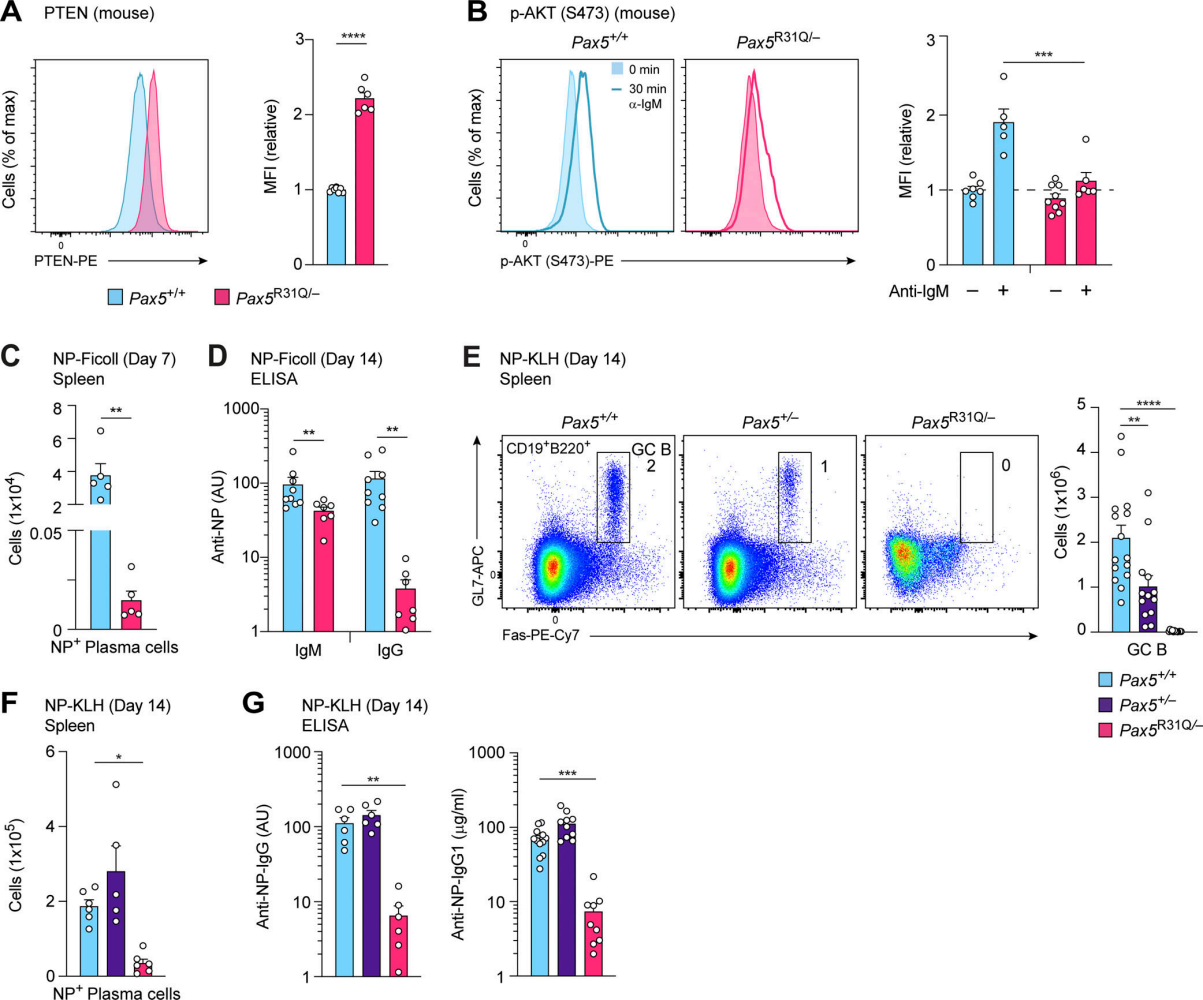
**Absence of B cell immune responses in *Pax5***^R31Q/E242^* and *Pax5*^**R31Q/−**^ **mice. (A)** PTEN expression in lymph node FO B cells was determined by intracellular staining with an anti-PTEN antibody (left). The median fluorescence intensity (MFI) of PTEN expression in *Pax5*^R31Q/−^ B cells is shown as mean value with SEM relative to the that of *Pax5*^+/+^ B cells (set to 1; left; *n* ≥ 6 per genotype). **(B)** Impaired PI3K-AKT signaling in *Pax5*^R31Q/−^ B cells. FO B cells from *Pax5*^R31Q/−^ and *Pax5*^+/+^ lymph nodes were either left untreated (−) or stimulated (+) for 30 min with anti-IgM before intracellular staining with an antibody detecting AKT phosphorylation at Ser473 (left). The MFI of phosphorylated AKT is shown as mean value with SEM relative to that of the *Pax5*^+/+^ B cells (set to 1; left; *n* ≥ 5 per genotype and condition). **(C)** NP^+^ plasma cells (NP_29_-PE^+^NP_14_-CGG-Alexa-Fluor-488^+^CD138^hi^TACI^hi^) in the spleen of the *Pax5*^+/+^, and *Pax5*^R31Q/−^ mice on day 7 after immunization with NP-Ficoll. Absolute cell numbers are shown as mean values with SEM (*n* = 5 per genotype). **(D)** Serum titers of NP-specific IgM and IgG antibodies 14 d after NP-Ficoll immunization. The antibody concentrations were measured by ELISA by using NP_24_-BSA–coated plates for capturing NP-specific IgM and IgG antibodies and are shown as mean values with SEM (*n* ≥ 7 per genotype). **(E)** GC B cell differentiation in the spleen of *Pax5*^+/+^, *Pax5*^+/−^, and *Pax5*^R31Q/–^ mice on day 14 after immunization with NP-KLH in alum. GC B cells (CD19^+^B220^+^Fas^+^GL7^+^) were identified by flow cytometry (left). Numbers refer to the percentage of cells in the indicated gate. Absolute cell numbers are shown as mean values with SEM (right; *n* ≥ 12 per genotype). **(F)** NP^+^ plasma cells in the spleen of the indicated genotypes on day 14 after NP-KLH immunization. Absolute cell numbers are shown as mean values with SEM (*n* ≥ 5 per genotype). **(G)** Serum titers of NP-specific IgG and IgG1 antibodies 14 d after NP-KLH immunization. The antibody concentrations were measured by ELISA by using NP_24_-BSA– or NP_7_-BSA–coated plates for capturing NP-specific IgG and IgG1 antibodies, respectively. NP-specific IgG1 concentrations (μg/ml) were determined relative to a standard NP-binding IgG1 monoclonal antibody. NP-specific IgG and IgG1 antibodies are shown as mean values with SEM (*n* ≥ 6 per genotype). Unpaired *t* test (A, C, and D), two-way ANOVA with Tukey’s multiple comparisons test (B), ANOVA with Dunnett’s or Dunnett’s T3 multiple comparisons test (E–G); *, P < 0.05; **, P < 0.01; ***, P < 0.001; ****, P < 0.0001. For detailed statistical information (A–G), see Table S6. Each dot (A–G) corresponds to one mouse.

Pax5 has recently been shown to fulfill an important function in mature B cells by promoting PI3K signaling by down-regulating the expression of the PTEN protein, a negative regulator of this pathway (Calderón et al., 2021). The PTEN protein was also upregulated in *Pax5*^R31Q/−^ FO B cells compared with *Pax5*^+/+^ B cells, which resulted in impaired phosphorylation of AKT at Ser473 after a 30-min stimulation with an anti-IgM antibody, as revealed by intracellular staining (Fig. 4, A and B). Analysis of EBV-immortalized B cells of the patient and a control revealed that PTEN expression was upregulated, and AKT phosphorylation at Ser473 was strongly reduced in B cells of the patient compared with the control (Fig. S1 F). We corroborated this result by stimulating naive mature B cells of the patient and a control for 30 min with anti-IgM, which induced phosphorylation of AKT at Ser473 in the control B cells, but not in B cells of the patient (Fig. S1 G). These data therefore demonstrated that the residual mature B cells of the patient were impaired in their function due to the observed PI3K signaling defect.

We next immunized *Pax5*^R31Q/−^ and control *Pax5*^+/+^ mice with the T cell–independent antigen Pl4-hydroxy-3-nitrophenylacetyl (NP)–conjugated Ficoll. NP-specific plasma cells were strongly reduced on day 7 after NP-Ficoll immunization in the spleen of *Pax5*^R31Q/−^ mice compared with *Pax5*^+/+^ mice (Fig. 4 C). Consistent with this finding, the anti–NP-IgG titer was 30-fold decreased in the serum of *Pax5*^R31Q/−^ mice relative to *Pax5*^+/+^ mice on day 14 after immunization, indicating that the immune response to NP-Ficoll is strongly impaired in *Pax5*^R31Q/−^ mice (Fig. 4 D). The anti–NP-IgM titer was, however, reduced only 2.3-fold in the serum of *Pax5*^R31Q/−^ mice (Fig. 4 D), which may reflect the similarly small decrease of IgM-producing B-1a cells in the spleen of these mice compared with *Pax5*^+/+^ mice (Fig. 3 B). Immunization with the T cell–dependent antigen NP-keyhole limpet hemocyanin (NP-KLH) in the adjuvant alum demonstrated that no germinal center (GC) B cells were generated in the spleen of *Pax5*^R31Q/−^ mice on day 14 after immunization (Fig. 4 E), similar to the absence of GC B cells in response to conditional *Pax5* deletion in mature B cells (Calderón et al., 2021). Consequently, NP-specific plasma cells were strongly reduced in the spleen of *Pax5*^R31Q/−^ mice (Fig. 4 F), which led to 17- and 19-fold lower levels of anti–NP-IgG and anti–NP-IgG1 in the serum of *Pax5*^R31Q/−^ mice compared with *Pax5*^+/+^ mice, respectively (Fig. 4 G). Finally, immunofluorescence analysis of spleen sections confirmed the absence of GC B cells together with a strong reduction of B cells in *Pax5*^R31Q/−^ mice (Fig. S1 H). In summary, these data demonstrated that the B cell immune responses to T cell–independent and T cell–dependent antigens were largely lost in the *Pax5*^R31Q/−^ mouse model, consistent with the patient’s diagnosis of hypogammaglobulinemia.

### Gene expression changes caused by selective DNA binding of the Pax5-R31Q protein

**Figure 5.**
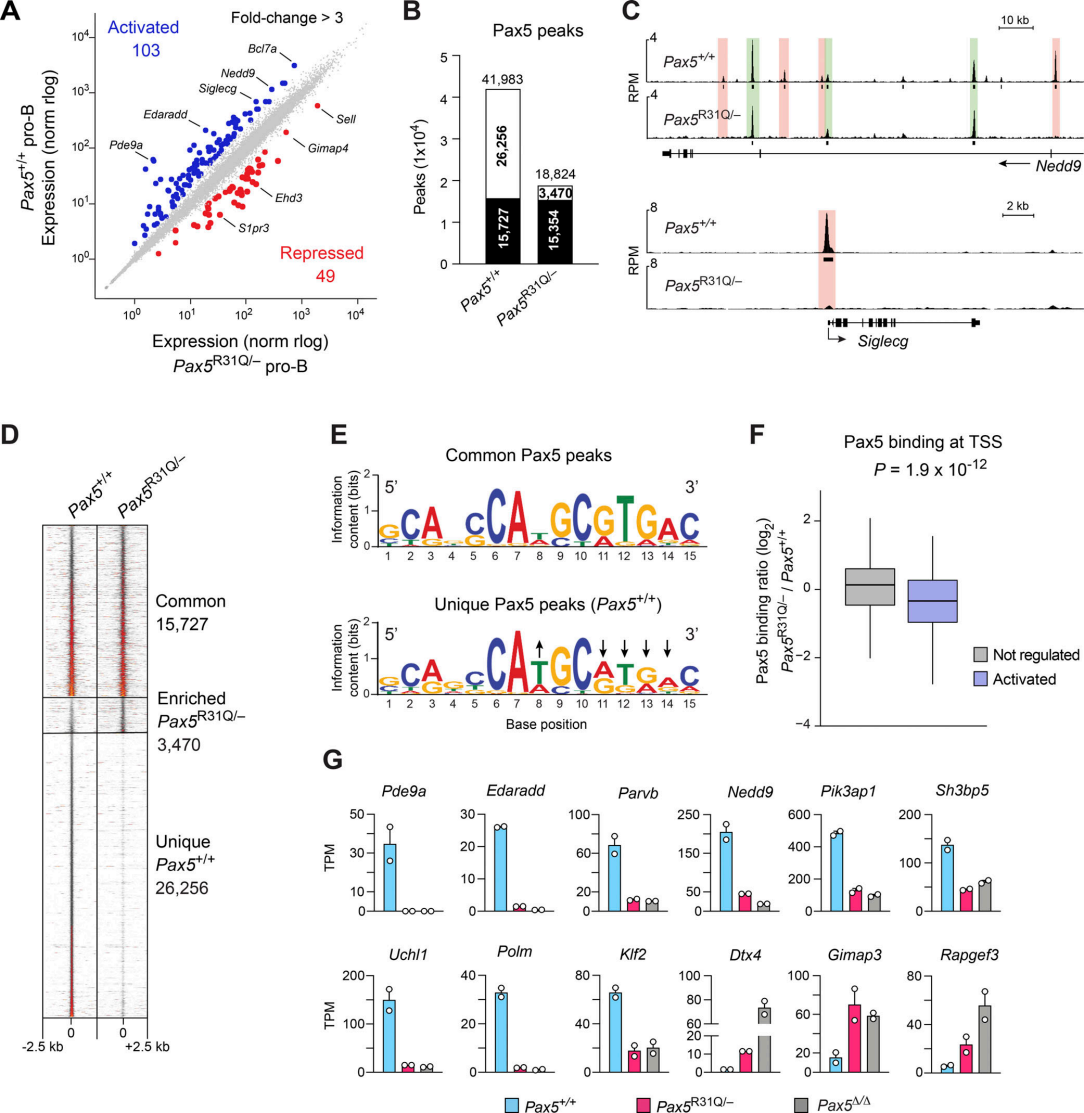
**Differential Pax5 binding and gene regulation in *Pax5***^**R31Q/−**^ **pro-B cells. (A)** Scatter plot of gene expression differences between ex vivo–sorted *Pax5*^+/+^ and *Pax5*^R31Q/−^ pro-B cells. The expression data of individual genes (dots) are plotted as mean normalized regularized logarithm (rlog) values and are based on two RNA-seq experiments per genotype (Table S3). Genes with an expression difference of >3-fold, an adjusted P value of <0.05, and TPM value of >5 (in at least one cell type) are colored in blue or red. **(B)** Overlap of Pax5 peaks between *Pax5*^+/+^ and *Pax5*^R31Q/−^ pro-B cells, as determined by ChIP-seq analysis of *Pax5*^+/+^ and *Pax5*^R31Q/−^ pro-B cells. Numbers refer to common (black) and unique (white) Pax5 peaks identified by MACS peak calling (P value <10^−10^). **(C)** Common (green) and unique (red) Pax5 peaks at *Nedd9* and *Siglecg*, visualized as reads per million (RPM). Horizontal bars indicate MACS-called peaks. **(D)** Density heatmaps of common and unique Pax5 peaks. **(E)** Pax5-binding motifs identified by de novo motif discovery analysis in common and unique peaks with *E* values of 8.1 × 10^−17^ and 2.4 × 10^−27^, respectively. Arrows point to nucleotide positions in the Pax5 motif of unique peaks that deviate from that of common peaks. **(F)** Correlation of gene activation with differential Pax5 binding at TSSs. The Pax5-binding difference at Pax5 peaks in 232 TSS regions of Pax5-activated (>2-fold) genes was calculated as a log_2_-fold ratio of the ChIP-seq normalized read CPM determined in *Pax5*^R31Q/−^ over *Pax5*^+/+^ pro-B cells (see Materials and methods). The distribution of the Pax5-binding difference at the TSS regions is shown for activated genes (>2-fold; blue) and nonregulated genes (expression >5 TPM, gray). The median (black line) and middle 50% (boxes) of the data and values within the 1.5 × interquartile range (whiskers) are shown; P value determined by two-tailed Student’s *t* test. **(G)** Expression of selected Pax5 target genes in *Pax5*^+/+^ (blue), *Pax5*^R31Q/−^ (red), and *Pax5*^−/−^ (*Vav*-Cre *Pax5*^fl/fl^; gray) pro-B cells, shown as mean TPM values of two RNA-seq experiments per genotype.

**Figure S2.**
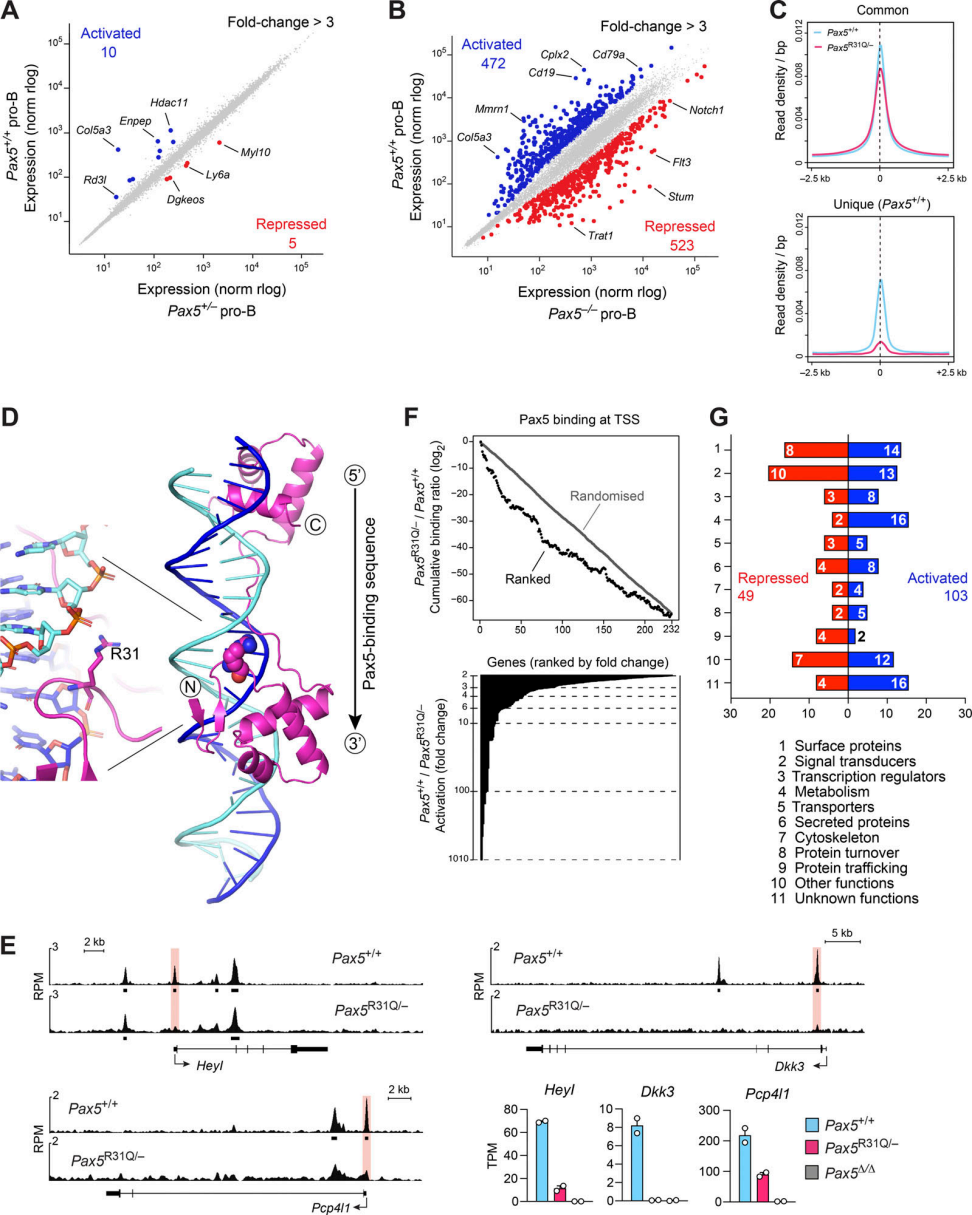
**Loss of Pax5 binding at the TSS correlates with loss of gene expression in *Pax5***^**R31Q/−**^ **pro-B cells. (A and B)** Scatter plot of gene expression differences between ex vivo–sorted *Pax5*^+/−^ and *Pax5*^+/+^ pro-B cells (A) as well as between ex vivo–sorted *Pax5*^−/−^ (*Vav*-Cre *Pax5*^fl/fl^) and *Pax5*^+/+^ pro-B cells (B). The expression data of individual genes (dots) are plotted as mean normalized regularized logarithm (rlog) values, based on two RNA-seq experiments per genotype. Genes with an expression difference of >3-fold, an adjusted P value of <0.05, and a TPM value of >5 (in at least one cell type) are colored in blue or red, corresponding to Pax5-activated or Pax5-repressed genes, respectively (Tables S4 and S5). **(C)** Binding density at Pax5 peaks identified by ChIP-seq in *Pax5*^+/+^ (blue) and *Pax5*^R31Q/−^ (red) pro-B cells and displayed from −1.5 to +1.5 kb relative to the summit of the Pax5 peaks that were common to both pro-B cell types or unique to *Pax5*^+/+^ pro-B cells (Fig. 3, B and D). **(D)** Crystal structure of the Pax5 paired domain bound to DNA (Garvie et al., 2001). The β-sheets (arrows) and α-helices are indicated together with Arg31 (R31), which interacts with the phosphate backbone in the minor groove of the DNA. The 5′-to-3′ orientation of the Pax5-binding sequence (Fig. 3 E) is indicated. For clarity, the ETS domain structure of Ets1, which is also part of the published x-ray structure, is not shown. **(E)** Correlation of loss of Pax5 binding at the TSS region and loss of mRNA expression of the indicated genes in *Pax5*^R31Q/−^ pro-B cells compared with *Pax5*^+/+^ pro-B cells. Horizontal bars indicate MACS-called Pax5 peaks. RPM, reads per million. **(F)** Correlation of gene activation with differential Pax5 binding at the TSS. The binding difference at Pax5 peaks in 232 TSS regions of Pax5-activated genes (>2-fold) was calculated as a log_2_-fold ratio of the ChIP-seq normalized read CPM determined in *Pax5*^R31Q/−^ over *Pax5*^+/+^ pro-B cells (see Materials and methods). The cumulative log_2_-fold ratios were plotted on the y axis for the 232 TSS regions of activated genes (black dots), which were ranked from high to low expression differences on the x axis (upper part). The fold activation of the ranked genes is shown below. The ranking of the activated genes was 100 times randomly shuffled to generate the randomized dataset of binding differences (gray). **(G)** Functional classification of the proteins encoded by the activated and repressed genes identified in *Pax5*^+/+^ versus *Pax5*^R31Q/−^ pro-B cells (Fig. 3 A and Table S3). Bar size indicates the percentage of activated or repressed genes in each functional class relative to the total activated or repressed genes, respectively. The gene number in each class is shown within the bar.

To investigate the molecular basis for the B cell developmental arrest in *Pax5*^R31Q/−^ mice, we performed RNA sequencing (RNA-seq) with ex vivo–sorted *Pax5*^+/+^ and *Pax5*^R31Q/−^ pro-B cells (Fig. 5 A). By considering mRNA expression differences of more than threefold between the two pro-B cell types, we identified 103 Pax5-activated and 49 Pax5-repressed genes that were deregulated in *Pax5*^R31Q/−^ pro-B cells (Fig. 5 A and Table S3). Notably, only 10 activated and 5 repressed genes were deregulated in *Pax5*^+/−^ pro-B cells compared with *Pax5*^+/+^ pro-B cells (Fig. S2 A and Table S4), indicating that the Pax5-R31Q protein must be responsible for the deregulation of most genes in *Pax5*^R31Q/−^ pro-B cells. These 103 activated and 49 repressed genes were, however, only a subset of the 472 activated and 523 repressed genes identified by comparing *Pax5*^+/+^ and *Pax5*^−/−^ pro-B cells (Fig. S2 B and Table S5), which raised the question whether binding of the Pax5-R31Q protein may be selectively lost at the subset of deregulated genes in *Pax5*^R31Q/−^ pro-B cells. By using a Pax5 paired domain antibody for chromatin immunoprecipitation coupled with deep sequencing (ChIP-seq) of short-term cultured *Pax5*^+/+^ and *Pax5*^R31Q/−^ pro-B cells, we identified 41,983 and 18,824 Pax5 peaks, respectively, with an overlap of 15,727 peaks between the two pro-B cell types (Fig. 5 B). Analysis of the *Nedd9* and *Siglecg* genes revealed that Pax5 binding at individual Pax5 sites was present either in both pro-B cell types or only in *Pax5*^+/+^ pro-B cells (Fig. 5 C). Density heatmaps of Pax5 binding confirmed this “all-or-nothing” phenotype, as the common Pax5 peaks had a similar Pax5-binding density in contrast to the strong binding difference observed at the unique peaks present in *Pax5*^+/+^ pro-B cells (Figs. 5 D and S2 C). De novo motif discovery analysis revealed that the Pax5-binding motif detected at the unique Pax5 peaks was considerably weakened compared with the respective Pax5 consensus motif of the common peaks at nucleotide positions 11–14 (Fig. 5 E) that are known to interact with the N-terminal part of the paired domain (Czerny et al., 1993; Garvie et al., 2001; Fig. S2 D). This finding is consistent with the fact that arginine 31 (R31) interacts with the phosphate backbone in the minor groove of the DNA, thus specifically enhancing DNA binding of the N-terminal module of the paired domain (Garvie et al., 2001; Fig. S2 D).

To investigate a correlation between the loss of Pax5 binding and gene expression, we focused our analysis on Pax5 peaks in the transcription start site (TSS) region of activated genes, as exemplified by the analysis of *Siglecg*, *Heyl*, *Dkk3*, and *Pcp4l1*. In all four cases, the Pax5 peak present at the TSS in *Pax5*^+/+^ pro-B cells was lost in *Pax5*^R31Q/−^ pro-B cells, which correlated with down-regulation of gene expression in *Pax5*^R31Q/−^ pro-B cells (Fig. 5, A and C; and Fig. S2 E). To systematically investigate the correlation between loss of Pax5 binding at the TSS and down-regulation of gene expression in *Pax5*^R31Q/−^ pro-B cells, we analyzed all activated genes that were more than twofold down-regulated in *Pax5*^R31Q/−^ pro-B cells and contained a Pax5 peak at their TSS in *Pax5*^+/+^ pro-B cells. The ratio of Pax5 binding between *Pax5*^R31Q/−^ and *Pax5*^+/+^ pro-B cells at the TSS of these activated genes was significantly reduced compared with that of expressed nonregulated genes (Fig. 5 F). We next explored whether the Pax5-binding difference at the TSS also correlated with the magnitude of gene expression difference. Plotting of the cumulative Pax5-binding ratio according to the ranked gene expression differences revealed that the loss of Pax5 binding at the TSS correlated with the degree of differential expression in *Pax5*^R31Q/−^ pro-B cells compared with *Pax5*^+/+^ pro-B cells (Fig. S2 F). This correlation was lost, however, by randomizing the ranking order of gene expression differences (Fig. S2 F). We therefore conclude that the selective DNA binding of Pax5-R31Q is responsible for the observed gene expression differences in *Pax5*^R31Q/−^ pro-B cells.

The deregulated genes code for proteins of distinct functional classes (Fig. S2 G). The three largest classes encoded by the activated genes are metabolic enzymes, cell surface proteins, and signal transducers, while signaling molecules and cell surface receptors were also prominently represented among the proteins encoded by the repressed genes (Fig. S2 G). As we could not explain the pro-B-to-pre-B cell developmental block by the loss or gain of function of a single deregulated gene, it is likely that the cumulative effect of several genes is responsible for the impaired B cell development in *Pax5*^R31Q/−^ mice. Activated Pax5 target genes, which are interesting in this regard (Fig. 5 G), code for the signaling molecules Pde9a, Edaradd, Pik3ap1, Uchl1, Parvb, Nedd9, Sh3bp5, transcription factor Klf2, and DNA polymerase μ (Polm) involved in V(D)J recombination (Bertocci et al., 2003), while repressed genes code for the signal transducers Dtx4, Gimap3, and Rapgef3. In summary, our molecular analyses uncovered the selective DNA-binding specificity of Pax5-R31Q as a cause for gene dysregulation and identified regulated genes contributing to the B cell developmental block in *Pax5*^R31Q/−^ mice.

### PAX5 deficiency leads to aberrant motor control and motor learning

Next, we hypothesized that the neurological and psychiatric phenotype of the patient is caused by the underlying *PAX5* mutations, particularly as *PAX5* haploinsufficiency has been associated with ASD (Gofin et al., 2022; Iossifov et al., 2012; O’Roak et al., 2014; Stessman et al., 2017). Furthermore, the patient presented with sufficient sensorimotor symptoms in daily life to consider the diagnosis of developmental coordination disorder (see patient description in Materials and methods; Table S2). We therefore predicted that the *Pax5*^R31Q/−^ and *Pax5*^+/−^ mouse models should show ASD-related and motor control deficits.

**Figure 6.**
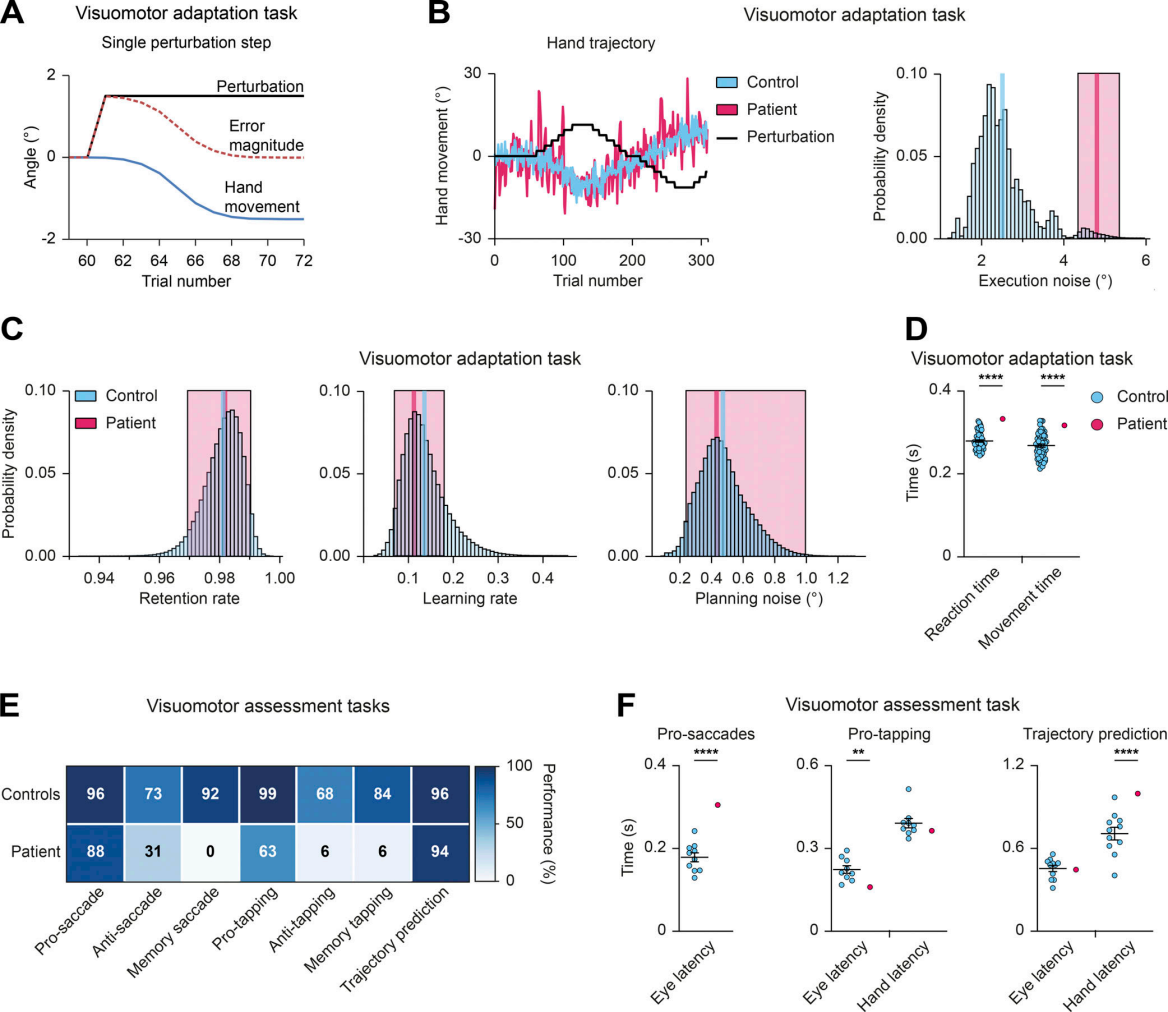
**PAX5 deficiency of the patient causes impaired motor control and learning. (A–D)** Visuomotor adaptation task (Video 1). **(A)** Schematic illustration of the hand movement angles and error angle magnitudes during a single adaptation step. As the movement angle approaches the inverse of the perturbation angle, the error magnitude decreases. In other words, participants compensate with their movements for the perturbation by minimizing the magnitude of the end-point error. **(B)** Visuomotor adaptation task. Hand movement traces of the patient (red) and a representative control (blue) during adaptation to the perturbation (black line). Right: Estimated execution noise (ε) parameters. 95% credible interval (CI) of the patient (red; σ_ε_ = 4.81, 95% CI 4.35–5.36) shown against the distribution of the controls (mean in blue; σ_ε_ = 2.02, 95% CI 1.90–2.14; *n* = 60). **(C)** Parameter estimates of the retention rate, learning rate, and planning noise, respectively. The patient’s mean value and 95% CI are shown in red (retention rate = 0.98, 95% CI 0.97–0.99; learning rate = 0.11, 95% CI 0.07–0.18; planning noise = 0.43, 95% CI 0.24–0.99). The mean value of the control group (*n* = 60) is shown in blue. The blue bars represent the density distribution for all controls. **(D)** Mean reaction and movement times of controls (blue; *n* = 60) and patient (red) across all trials. The reaction time was defined as the time from the appearance of the target until movement initiation. The movement time was defined as the time from movement initiation until movement end. **(E)** Data summary of the visuomotor assessment tasks (Video 2). Performance matrix of the indicated tasks is shown for the patient and controls (*n* ≥ 7) with percentage of correct trials, using the mean value of the controls. **(F)** Visuomotor assessment tasks. Time reflecting eye and hand latency of the patient and controls (*n* ≥ 9) during the pro-saccade, pro-tapping, and trajectory prediction trials, respectively (Video 2). Two-tailed one-sample *t* test (D and F); **, P < 0.01; ****, P < 0.0001. For detailed statistical information (D and F), see Table S6. Each dot represents one individual (D and F).

To this end, we first obtained detailed information on the patient’s sensorimotor skills by subjecting him to the visuomotor adaptation (Krakauer et al., 1999) and the visuomotor assessment (Manns et al., 2000) tasks. In the visuomotor adaptation task, the visual feedback differs from the actual arm movement, which requires continuous manual compensation by adjusting the angle of the arm movement to adapt to these deviations (Video 1 and Fig. 6 A). The patient was able to complete the task (Fig. 6, B and C), but showed a high degree of execution noise in his movements (van der Vliet et al., 2018; Fig. 6 B) and delayed reaction times (Fig. 6 D), which is consistent with the previously observed motor deficits (see patient description in Materials and methods).

**Video 1.** The visuomotor adaptation task used to generate the data of Fig. 6, A–D.

The visuomotor assessment tasks consist of seven assays that measure eye and hand movement kinematics, coordination, and memory (Video 2). The patient’s performance was within the normal range for pro-saccade, pro-tapping, and trajectory prediction tests (Fig. 6 E). However, eye movements were delayed in the pro-saccade task, and the patient had clear delays in initiating the hand movement towards the correct target in the trajectory prediction task (Fig. 6 F), indicating a visuomotor integration problem. In the pro-tapping task, the patient showed shorter eye latency than controls (Fig. 6 F). In addition, his performance was poor in the anti-saccade and anti-tapping tests (Fig. 6 E). In the incorrect trials, the patient looked towards the target and performed corrective saccades toward the opposite direction in >50% of the trials, suggesting that, although he understood the task, he was unable to inhibit the reflex toward the presented stimulus. His performance in the memory-saccade and memory-tapping tasks was too poor to quantify any latencies.

**Video 2.** The visuomotor assessment task used to generate the data of Fig. 6, E and F.

**Figure 7.**
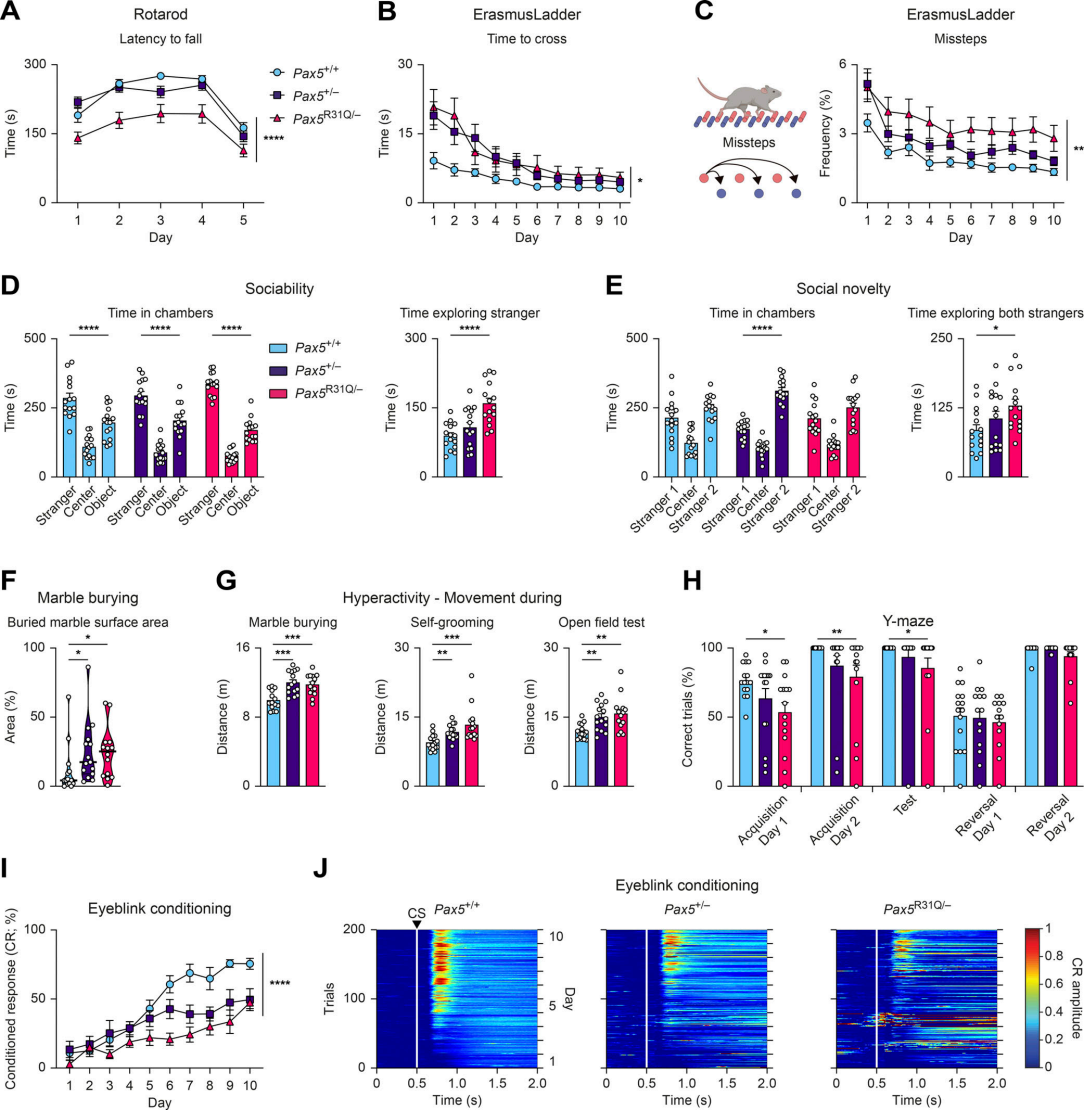
***Pax5* mutant mice exhibit impaired motor control and learning, as well as ASD-related deficits.** All behavioral experiments were performed with *Pax5*^+/+^, *Pax5*^+/−^, and *Pax5*^R31Q/−^ mice (*n* ≥ 9 per genotype) at the age of 2–4 mo. **(A)** Rotarod test. The latency to fall (acceleration: days 1–4, 40 revolutions per min; day 5, 80 revolutions per min). **(B)** Mean time per trial to cross the ErasmusLadder (Vinueza Veloz et al., 2015). **(C)** Mean frequency of missteps as percentage of total steps on the ErasmusLadder. **(D and E)** Three-chamber sociability and social novelty tests (Fig. S3 C). **(D)** Sociability test. Left: Total time spent in the chambers. Right: Total time exploring “stranger 1” mouse. **(E)** Social novelty test. Left: Total time spent in chambers after introduction of a second “stranger 2” mouse. Right: Total time actively exploring both stranger mice. **(F)** Marble burying test. Percentage of marble surface area that is covered by bedding. **(G)** Hyperactivity profile. Distance covered during marble burying, self-grooming, and open field tests. **(H)** Percentage of correct trials in the water Y-maze test. **(I and J)** Eyeblink conditioning (Video 3). **(I)** Percentage of conditioned responses across the entire experiment for CS-only trials. **(J)** Heatmaps of group average amplitude of conditioned responses during the CS-only trials (white lines and the arrowhead denote the CS onset). All graphs show mean values with SEM, except for the violin plots (F), indicating median values with quartiles (*n* ≥ 9 per genotype). Two-way repeated-measures ANOVA with Dunnett’s multiple comparisons test (A–C and I), two-way ANOVA with Tukey’s multiple comparisons test (D and E), ANOVA with Dunnett’s multiple comparisons test (G and H), Kruskal–Wallis test with Dunn’s multiple comparisons test (F); *, P < 0.05; **, P < 0.01; ***, P < 0.001; ****, P < 0.0001. For detailed statistical information (A–I), see Table S6. Each dot (D–H) corresponds to one mouse.

**Figure S3.**
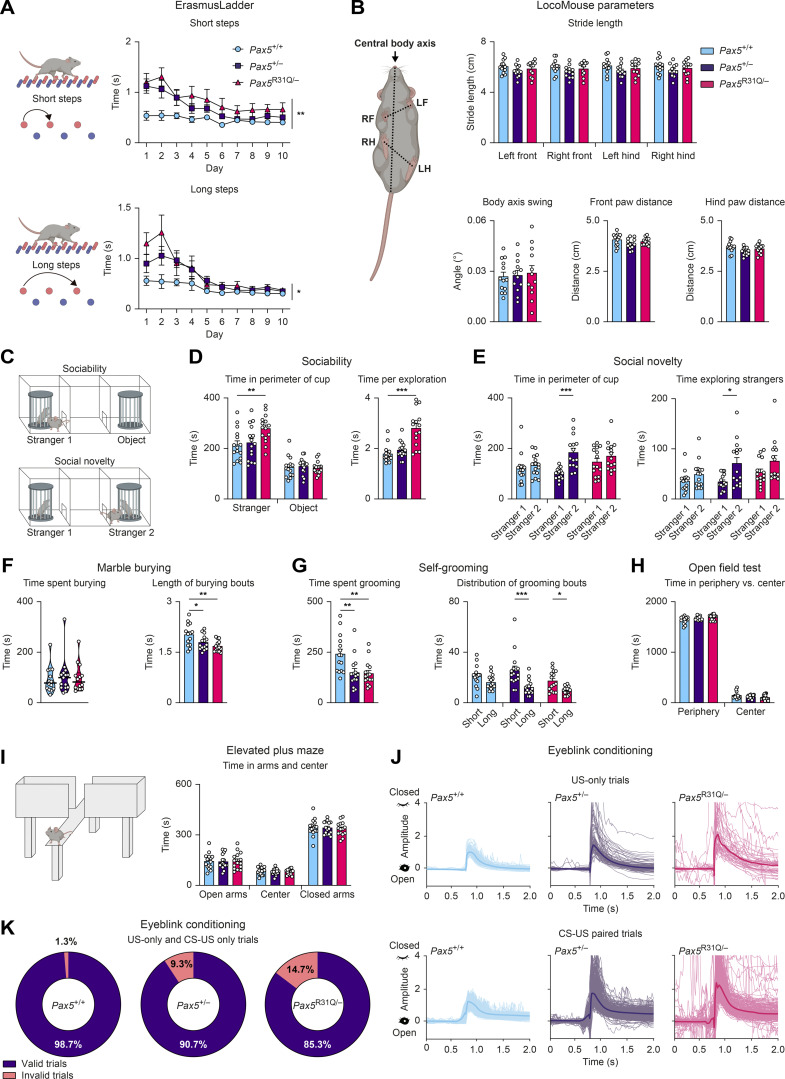
***Pax5* mutant mice display poor motor coordination and ASD-related behavioral abnormalities.** All behavioral experiments (A–K) were performed with *Pax5*^+/+^, *Pax5*^+/−^, and *Pax5*^R31Q/−^ mice (*n* ≥ 9 per genotype) at the age of 2–4 mo. **(A)** ErasmusLadder. The time required for short or long steps is shown as mean value with SEM. **(B)** LocoMouse. Left: The illustration indicates the measured parameters. Top right: Stride length for each individual paw. Bottom left: The body axis swing reflects the angular difference of the central body axis between the placement of the right-front (RF) and left-hind (LH) diagonal paws and the left-front (LF) and right-hind (RH) diagonal paws. Bottom middle-right: The distances between the front paws (middle) or hind paws (right) during locomotion, as indicated by the dotted line in the cartoon. **(C–E)** Three-chamber sociability and social novelty test. **(C)** Illustration of the setup with a single “stranger 1” mouse in one cup (sociability test) or with two stranger mice (“stranger 1 and 2”) in separate cups (social novelty test). **(D)** Sociability test. Left: Total time spent in the perimeter of the cup with stranger 1 mouse or the empty cup (object). Right: Mean length of bouts actively exploring stranger 1 mouse. **(E)** Social novelty test. Left: Total time spent in the perimeter of the cup with stranger 1 or stranger 2. Right: Mean length of bouts actively exploring stranger mouse 1 and 2. **(F)** Marble burying test. Left: Total time spent burying. Right: Mean length of burying bouts. **(G)** Self-grooming behavior. Left: Total time spent grooming. Right: Mean time of short and long grooming bouts, respectively. Short bouts were defined as 0–3.5 s, long bouts as >3.5 s. **(H)** Open field test. Total time spent at the periphery or in the center of the arena. **(I)** Elevated plus maze. Left: The illustration indicates the closed and open arms of the platform. Right: Time spent in the open and closed arms as well as in the center. **(J and K)** Eyeblink conditioning. **(J)** Examples of individual traces during US-only (top) and CS-US (bottom) trials (see Video 3 for explanation of the trial types). **(K)** Quantification of valid and invalid trials during US-only and CS-US–only trials (see Materials and methods for detailed description of the experiment). The data (B and D–I) are shown as mean values with SEM (*n* ≥ 12 per genotype). Two-way repeated-measures ANOVA with Dunnett’s multiple comparisons test (A), ANOVA with Dunnett’s or Dunnett’s T3 multiple comparisons test (B, F, and G), two-way ANOVA with Tukey’s multiple comparisons test (D, E, H, and I); *, P < 0.05; **, P < 0.01; ***, P < 0.001. For detailed statistical information (B and D–I), see Table S6. Each dot represents one mouse.

We next analyzed these findings in the mouse model. *Pax5*^R31Q/−^ mice demonstrated substantial motor performance deficits in the Rotarod assay compared with *Pax5*^+/−^ and *Pax5*^+/+^ mice (Fig. 7 A). In the ErasmusLadder test (Vinueza Veloz et al., 2015), both *Pax5*^R31Q/−^ and *Pax5*^+/−^ mice demonstrated impaired performance as illustrated by the prolonged time required to traverse the ErasmusLadder (Fig. 7 B), the increased frequency of missteps (Fig. 7 C), and the increased time performing short and long steps (Fig. S3 A). Although both mutant mouse strains exhibited clear motor performance deficits, we did not observe major gait abnormalities in the LocoMouse paradigm (Machado et al., 2015). Both mutants showed similar stride lengths, normal center body axis swing, and comparable distances measured between the front and hind paws during locomotion (Fig. S3 B), thus excluding ataxia as a confounding phenotype. In summary, similar motor performance and motor learning deficits were observed in both the patient and the *Pax5*^R31Q/−^ mouse model.

### *Pax5*^R31Q/−^ and *Pax5*^+/−^ mice exhibit aberrant social and stereotypical behavior, hyperactivity, and cognitive impairments

To understand the role of PAX5 deficiency in ASD pathogenesis, we next assessed potential social deficits in the three-chamber social interaction assay (Fig. S3 C). Whereas both *Pax5* mutant mouse strains demonstrated a preference for the chamber with the stranger mouse similar to that of wild-type mice (Fig. 7 D), *Pax5*^R31Q/−^ mice spent more time in the perimeter of the cup containing the stranger mouse (Fig. S3 D). In addition, *Pax5*^R31Q/−^ mice spent more time actively exploring the stranger mouse, and their exploration bouts lasted significantly longer compared with controls (Figs. 7 D and S3 D). These findings unveiled hypersociability as a prominent trait of the *Pax5*^R31Q/−^ mouse, which may reflect the social disinhibition of the patient (see patient description in Materials and methods).

In the social novelty phase of the assay (Fig. S3 C), the *Pax5*^R31Q/−^ and control *Pax5*^+/+^ mice showed no bias for the newly introduced, second stranger mouse (Fig. 7 E). In contrast, *Pax5*^+/−^ mice spent more time in the chamber (Fig. 7 E) as well as in the perimeter of the cup with the novel stranger (Fig. S3 E), which was also reflected by the higher total time actively exploring the novel stranger (Fig. S3 E). Notably, the *Pax5*^R31Q/−^ mice spent more time actively exploring both stranger mice (Fig. 7 E), thereby reconfirming hypersociability as a distinctive feature of this genotype.

We next assessed stereotypical traits in the marble-burying test and by observing self-grooming behavior. Both *Pax5* mutants buried more marbles compared with controls (Fig. 7 F). However, there was no difference in the total time spent burying, which could be explained by the shorter length of the burying bouts (Fig. S3 F). Furthermore, both *Pax5* mutants spent less time grooming, with a shift towards shorter grooming bouts (Fig. S3 G). Of note, both *Pax5* mutants covered a greater distance during these experiments compared with wild-type controls (Fig. 7 G). We did not observe any differences in exploratory behavior in the open field test and on the elevated plus maze (Fig. S3, H and I), thus excluding anxiety as a potential explanation for the observed hyperactivity. Furthermore, both *Pax5* mutants also covered a greater distance in the open field test, thereby confirming that hyperactivity is a distinctive phenotype of both mutants in all three assays, allowing mice to freely roam open spaces (Fig. 7 G). These findings are consistent with the restlessness that was reported for the patient (see patient description in Materials and methods).

Subsequently, we tested cognitive abilities by assessing spatial and reversal learning in the Y-maze. *Pax5*^R31Q/−^ mice displayed impaired spatial learning as illustrated by the increased number of trials required to find the location of the platform during the acquisition phase and on the test day of the experiment (Fig. 7 H). In fact, we extended the acquisition phase of the Y-maze assay to 2 d, because of the severe learning deficits of the *Pax5*^R31Q/−^ mice, which is consistent with the observed cognitive impairment of the patient. We did not observe impaired reversal learning after the location of the platform was reversed.

Lastly, we assessed motor learning by means of the eyeblink conditioning paradigm, in which mice learn to close their eye to a light pulse (conditioned stimulus [CS]) in anticipation of an air puff to the cornea (unconditioned stimulus [US]; Video 3). We observed impaired motor learning as illustrated by the reduced frequency and amplitude of conditioned responses in both *Pax5* mutant mice (Fig. 7, I and J). *Pax5*^R31Q/−^ mice also displayed gross impairments in timing the eye closure when presented with the US, demonstrating deficits in cerebellar-dependent sensorimotor learning (Fig. S3, J and K).

**Video 3.** The eyeblink conditioning test used to generate the data of Fig. 7, I and J.

**Figure S4.**
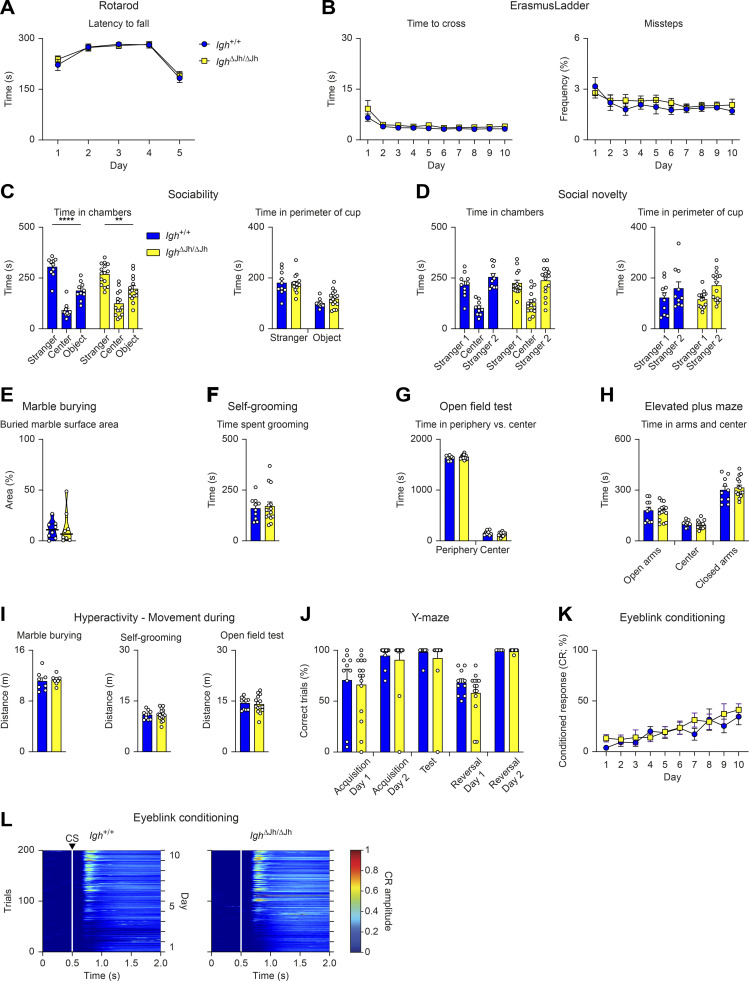
**B cell–deficient *Igh***^**∆Jh/∆Jh**^ **mice show no behavioral abnormalities.** All behavioral experiments (A–L) were performed with control *Igh*^+/+^ mice (blue) and *Igh*^∆Jh/∆Jh^ (yellow) littermates (*n* ≥ 8 per genotype) at the age of 2–4 mo. **(A)** Rotarod assay. Latency to fall with a maximum acceleration of 40 revolutions per min on day 1–4 and 80 revolutions per min on day 5. **(B)** ErasmusLadder. Left: Mean time to cross the ladder per trial. Right: Mean frequency of missteps as percentage of total steps. **(C and D)** Three-chamber sociability and social novelty test. **(C)** Sociability test. Left: Total time in the chamber containing a cup with a “stranger 1” mouse, the center, or in the chamber with an empty cup (object). Right: Total time in the perimeter of the cup with stranger 1 mouse or the empty cup (object). **(D)** Social novelty test. Left: Total time in the chambers after introduction of a second “stranger 2” mouse. Right: Total time spent in the perimeter of the cup with stranger 1 or stranger 2. **(E)** Marble burying test. Percentage of marble surface area that is covered by bedding. **(F)** Self-grooming behavior. Total time spent grooming. **(G)** Open field test. Total time spent at the periphery or in the center of the arena. **(H)** Elevated plus maze. Time spent in the open and closed arms as well as in the center. **(I)** Hyperactivity profile. Distance covered during marble burying, self-grooming, and open field tests. **(J)** Water Y-maze assay. Percentage of correct trials during the acquisition, test, and reversal phases of the assay. During the reversal phase, the platform is placed in the opposite arm to assess reversal learning. **(K and L)** Eyeblink conditioning, as described in Video 3. **(K)** Percentage of conditioned responses (eye closure upon light pulse) across the entire experiment for CS-only trials. **(L)** Heatmaps displaying the mean amplitude of the conditioned response during the CS-only trials across the entire 10-d experiment for all mice of each genotype (white lines and the arrowhead denote the CS onset). All graphs show mean values with SEM, except for the violin plots (E), indicating median values with quartiles (*n* ≥ 8 per genotype). Two-way repeated measures ANOVA with Šídák’s multiple comparisons test (A, B, and K), two-way ANOVA with Tukey’s multiple comparisons test (C, D, G, and H), Mann–Whitney test (E), unpaired *t* test (F, I, and J); **, P < 0.01; ****, P < 0.0001. For detailed statistical information (A–K), see Table S6. Each dot (C–J) represents one mouse.

To exclude a confounding effect of B cell immunodeficiency on the neurological deficits of *Pax5*^R31Q/−^ mice, we repeated the same test battery with *Igh*^∆Jh/∆Jh^ mice that cannot recombine the immunoglobulin heavy-chain locus, thus leading to a complete block of B cell development (Gu et al., 1993). We did not observe behavioral differences between *Igh*^∆Jh/∆Jh^ and control *Igh*^+/+^ mice in any of the aforementioned assays (Fig. S4, A–L), thereby excluding any potential effect of B cell deficiency on the neurological phenotype of the *Pax5*^R31Q/−^ mouse. Hence, this phenotype is caused by loss of the brain-intrinsic function of Pax5.

### Biallelic *PAX5* mutations cause aberrant cerebellar foliation

**Figure 8.**
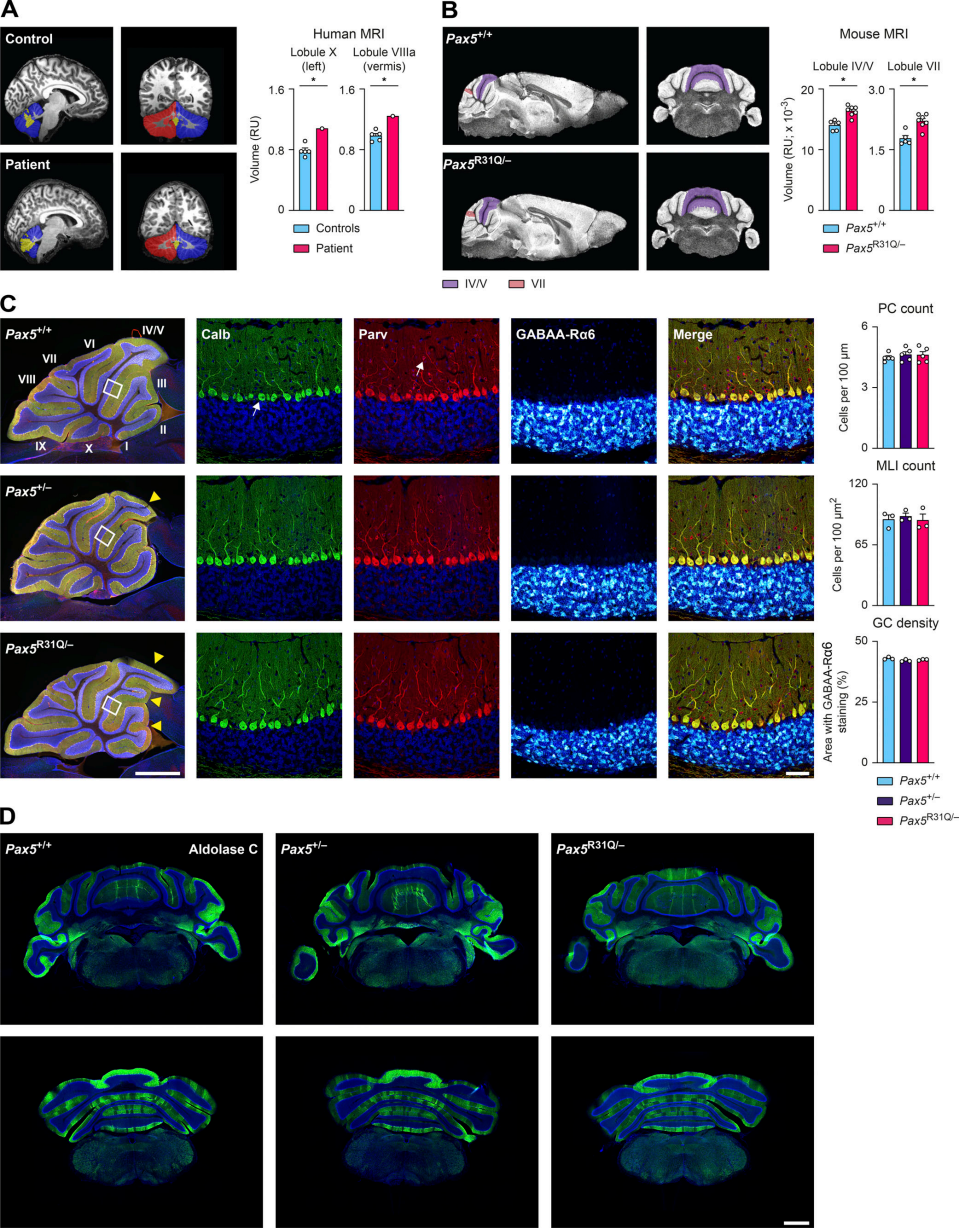
**PAX5 deficiency causes aberrant foliation of the cerebellum. (A)** Left: Sagittal and coronal MRI brain scans of the patient and a representative control. The left (blue) and right (red) hemispheres and the vermis (yellow) of the cerebellum are indicated. Right: Volumetric quantification of the lobules X (left hemisphere) and VIIIa (vermis) of the patient and five controls. **(B)** Left: Sagittal and coronal MRI brain scans of adult *Pax5*^+/+^ and *Pax5*^R31Q/−^ mice. Right: Quantification of lobules IV/V and VII. **(C)** Immunofluorescent staining of sagittal brain sections from adult mice of the indicated genotypes. Left: The entire section of the cerebellum is shown after combined the staining with antibodies detecting calbindin (Calb, green), parvalbumin (Parv, red), and GABAA-R6α (white) together with DAPI (blue). The different lobules of the vermis are indicated by roman numbers. The altered foliation pattern in the vermis of *Pax5*^+/−^ and *Pax5*^R31Q/−^ brains is shown by yellow arrowheads. The scale bar denotes 1 mm. Center: Magnification of the inset regions shown in the overview sections (to the left) displays the individual staining of calbindin (arrow pointing to an individual Purkinje cell), parvalbumin (arrow pointing to an individual molecular layer interneuron), and GABAA-R6α (white) in combination with DAPI (blue). All stainings are merged in the image shown to the right. The scale bar denotes 50 μm. Right: Bar graphs show the density (cell numbers across monolayer or across area, respectively) of Purkinje cells (PC; calbindin expression), molecular layer interneurons (MLI; parvalbumin expression), and granule cells (GC; GABAA-R6α expression) in lobule IV/V (data obtained from midline sections of three mice per genotype for the GC and MLI quantification and five mice per genotype for the PC quantification). **(D)** Coronal cerebellar sections of adult mice of the indicated genotypes stained with an aldolase C–specific antibody (green) combined with DAPI (blue). One of three experiments is shown (*n* = 3 per genotype). Scale bars denote 1 mm (C, overview); 50 μm (C, magnification); and 1 mm (D). The data (A–C) are shown as mean values with SEM (*n* = 5 for controls in A, *n* ≥ 5 per genotype in B and *n* ≥ 3 per genotype in C). Two-tailed one-sample *t* test (A), unpaired *t* test with false discovery rate correction (B), ANOVA with Dunnett’s multiple comparisons test (C); *, P < 0.05. For detailed statistical information (A–C), see Table S6. Each dot represents one individual (A) or a single mouse (B and C).

Given the aberrant neuroanatomy of the midbrain and cerebellum in *Pax5*^−/−^ mice (Urbánek et al., 1994), we next investigated the cerebellar anatomy of the patient. Ultra-high-field magnetic resonance imaging (MRI) of the brain of the patient and age- and gender-matched controls revealed that the volumes of several cerebellar structures, such as the left lobule X and the vermal lobule VIIIa, were significantly altered in the patient compared with controls (Fig. 8 A and statistical data of Fig. 8 A in Table S6). Similarly, high-resolution MRI of the brains of *Pax5*^R31Q/−^ and control *Pax5*^+/+^ mice revealed significant differences in the volume of the vermal lobules IV/V and VII (Fig. 8 B). Moreover, abnormal foliation of the vermal lobules in *Pax5*^R31Q/−^ mice was consistently seen in all histological experiments (Fig. 8 C, left). These findings, however, were accompanied neither by differences in the density of Purkinje cells, molecular layer interneurons, and granule cells (Fig. 8 C, middle and right) nor by an abnormal cerebellar topography, as illustrated by the normal parasagittal pattern of aldolase C–positive and –negative zones and stripes in the *Pax5* mutant brains (Fig. 8 D). We conclude therefore that biallelic *PAX5* mutations lead to altered foliation of the cerebellum.

### Biallelic *PAX5* mutations lead to hypoplasia of the substantia nigra (SN) and ventral tegmental area (VTA) with loss of GABAergic neurons

**Figure 9.**
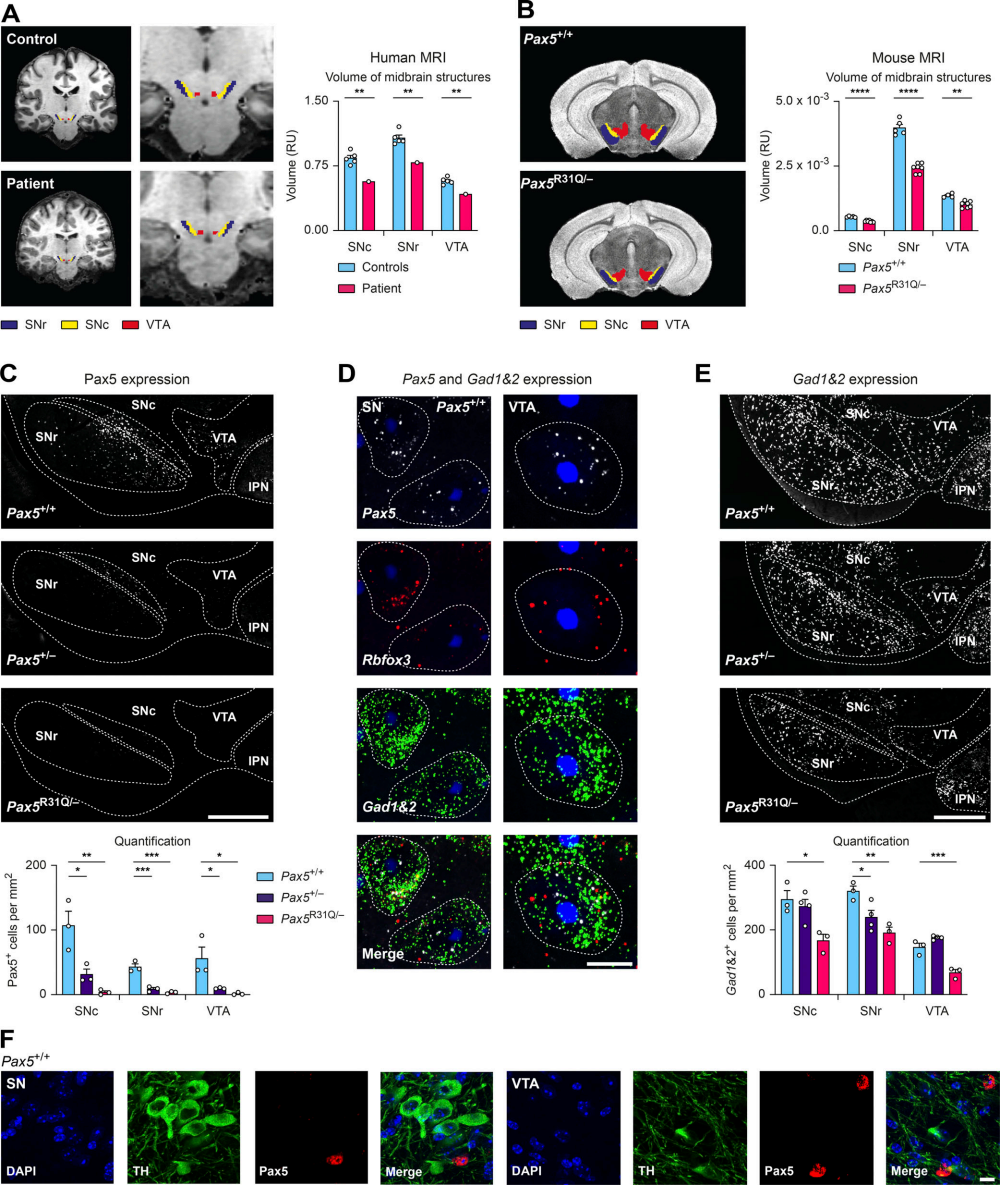
**PAX5 deficiency causes hypoplasia of the SN and VTA with loss of GABAergic neurons. (A)** Left: Coronal MRI brain scans of the patient and a representative control. The SNr (blue), SNc (yellow), and VTA (red) are indicated. Right: Volumetric quantification of the three midbrain regions. **(B)** Left: Coronal MRI brain scans of adult *Pax5*^+/+^ and *Pax5*^R31Q/−^ mice. Right: Quantification of the three midbrain regions. **(C)** Top: Anti-Pax5 staining (white) of *Pax5*^+/+^, *Pax5*^+/−^, and *Pax5*^R31Q/−^ adult mouse brain sections. Dotted lines delineate the SNr, SNc, VTA, and interpeduncular nucleus (IPN). Bottom: Quantification of Pax5-expressing cells. **(D and E)** smRNA-FISH staining. **(D)** SN- and VTA-containing section of an adult wild-type mouse with *Pax5* (white), *Rbfox3* (red), *Gad1*/*Gad2* (green), and DAPI (blue) staining. Data are representative of three experiments. Dotted lines delineate cell boundaries demarcated by *Gad1*/*Gad2* mRNA staining. **(E)** Top: *Gad1*/*Gad2* mRNA (white) of *Pax5*^+/+^, *Pax5*^+/−^, and *Pax5*^R31Q/−^ adult mouse brains. Dotted lines delineate SNr, SNc, VTA, and IPN. Bottom: Quantification of *Gad1*/*Gad2-*expressing cells (*n* ≥ 3 mice per indicated genotype). **(F)** Immunofluorescent staining of coronal sections of an adult wild-type midbrain with antibodies detecting tyrosine hydroxylase (TH; green) and Pax5 (red) in combination with DAPI (blue). The data are representative of three experiments with different wild-type mice. Scale bars: 500 μm (C and E); 10 μm (D); 20 μm (F). RU, relative units. Data (A–C and E) are shown as mean values with SEM (*n* = 5 for controls in A, *n* ≥ 4 per genotype for B, and *n* ≥ 3 per genotype for C and E). Two-tailed one-sample *t* test with false discovery rate (FDR) correction (A), unpaired *t* test with FDR correction (B), ANOVA with Dunnett’s multiple comparisons test (C and E); *, P < 0.05; **, P < 0.01; ***, P < 0.001; ****, P < 0.0001. For detailed statistical information (A–C and E), see Table S6. Each dot represents one individual (A) or a single mouse (B, C, and E).

Given the previously published expression of *Pax5* in the murine midbrain (Stoykova and Gruss, 1994; Urbánek et al., 1994), we investigated the neuroanatomy of the patient’s midbrain. Within the midbrain of the patient, we observed hypoplasia of several midbrain and subcortical structures, which was most notable in the SN pars compacta (SNc) and pars reticularis (SNr) as well as in the VTA, compared with age- and gender-matched controls (Fig. 9 A). These three regions were also significantly reduced in the midbrain of *Pax5*^R31Q/−^ mice compared with *Pax5*^+/+^ mice (Fig. 9 B). Hence, the same neuroanatomic defects were observed in both the patient and corresponding mouse model.

We next investigated the expression pattern of Pax5 in the adult mouse brain by immunofluorescence analysis. Pax5 protein expression was mostly confined to the midbrain in *Pax5*^+/+^ mice, where it was observed in several regions, including the SN and VTA (Fig. 9 C). Single-cell RNA-seq analysis of the developing human midbrain has previously revealed *PAX5* expression in GABAergic midbrain neurons (La Manno et al., 2016). Using single-molecule RNA (smRNA) FISH analysis of wild-type adult mouse brains, we could demonstrate that *Pax5* mRNA was strongly expressed in GABAergic neurons within the midbrain, as shown by the colocalized expression of *Pax5*, the GABAergic marker genes *Gad1* and *Gad2*, and the neuronal marker gene *Rbfox3* (NeuN; Fig. 9 D). In the SN and VTA, Pax5 was not expressed in dopaminergic cells, as shown by the mutually exclusive expression of Pax5 and tyrosine hydroxylase in these brain regions (Fig. 9 F).

We hypothesized that the hypoplasia of the SN and VTA in *Pax5*^R31Q/−^ mice might be caused by the loss of Pax5-positive GABAergic neurons. To this end, we quantified the expression of Pax5 in both midbrain regions of *Pax5*^+/+^, *Pax5*^+/−^, and *Pax5*^R31Q/−^ mice and observed reduced Pax5 expression with increasing severity of the *Pax5* mutations (Fig. 9 C). The reduced Pax5 expression in the SN of the *Pax5* mutant mice was accompanied by an increasing loss of GABAergic neurons in the SNr, which also correlated with the severity of the *Pax5* mutations (Fig. 9 E). In the SNc and VTA, only *Pax5*^R31Q/−^ mice showed a loss of GABAergic neurons (Fig. 9 E). Together, these data demonstrated that the impaired Pax5 function in *Pax5*^R31Q/−^ mice caused the loss of Pax5-expressing GABAergic neurons in the SN and VTA.

### Pax5 is expressed in cerebellar progenitor cells and during neurogenesis of GABAergic midbrain neurons

**Figure S5.**
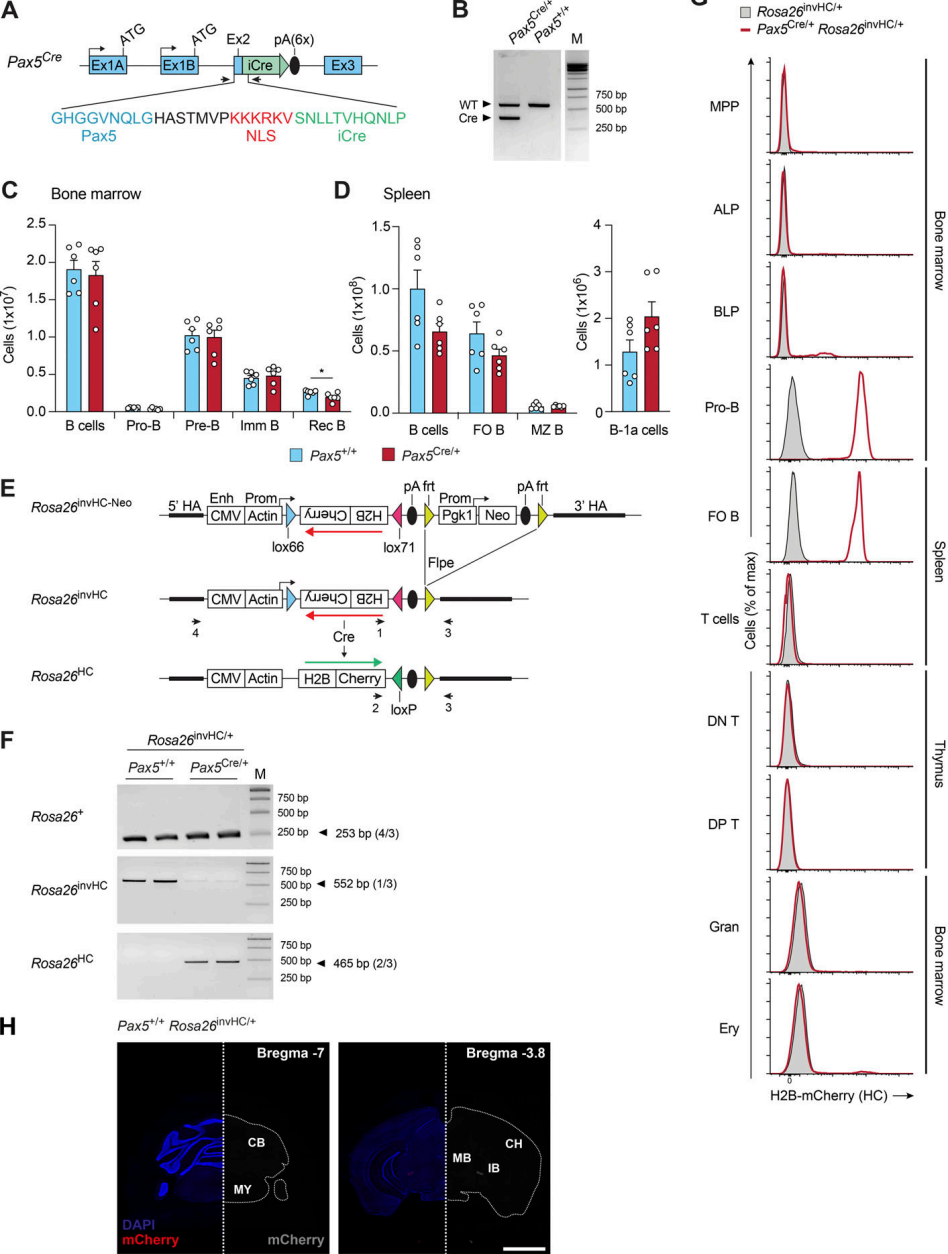
**Generation and characterization of the *Pax5***^**Cre**^ **and *Rosa26***^**invHC**^ **alleles. (A)** Generation of the *Pax5*^Cre^ allele. The *Pax5*^Cre^ allele was generated by in-frame insertion of a codon-improved (i) Cre gene, linked to six copies of a synthetic poly(A) site (Levitt et al., 1989), after the ninth codon of *Pax5* exon 2. Single-stranded DNA of this Cre gene insertion, flanked by 250-bp *Pax5* homology regions, was injected with Cas9 protein and *Pax5* exon 2-specific sgRNAs (Table S7) into mouse zygotes (Miura et al., 2018) to generate the *Pax5*^Cre/+^ mouse strain. Arrows indicate primers 1 and 2 (Table S7) for PCR genotyping of the *Pax5*^Cre^ allele. NLS, nuclear localization sequence. **(B)** PCR genotyping of tail DNA from a *Pax5*^Cre/+^ mouse. The PCR fragments indicative of the *Pax5*^Cre^ (353-bp) and WT *Pax5* (558-bp) alleles are shown with a DNA marker (M). **(C and D)** Flow-cytometric analysis of B cell development in the bone marrow (C) and spleen (D) of *Pax5*^Cre/+^ (red) and control *Pax5*^+/+^ (blue) mice. Absolute numbers of total B cells and the indicated B cell types (see Materials and methods) are shown as mean values with SEM (*n* = 6 per genotype). Imm, immature; Rec, recirculating. **(E)** Generation of the *Rosa26*^invHC^ allele. DNA sequences encoding the human histone H2B fused in frame to mCherry (HC) were inserted between convergent *lox*66 and *lox*71 sites (Anastassiadis et al., 2010) in inverted (inv) orientation downstream of a CMV enhancer and chicken actin promoter into a *Rosa26* targeting vector containing a *frt*-flanked *Pgk1* promoter-neomycin resistance (Neo^r^) gene cassette, which was subsequently eliminated by Flpe-mediated recombination to generate the *Rosa26*^invHC^ allele. The *Rosa26*^HC^ allele was generated by Cre-mediated reversal of the inverted HC insert of the *Rosa26*^invHC^ allele. HA, homology arm. The PCR primers (1–4) are shown. **(F)** PCR genotyping of the *Rosa26*^+^, *Rosa26*^invHC^, and *Rosa26*^HC^ alleles. DNA from sorted splenic B cells was used for amplification of PCR fragments of the indicated sizes with the indicated primer pairs (Table S7). **(G)** Flow-cytometric analysis of hematopoietic cell types from the bone marrow, spleen, and thymus of *Pax5*^Cre/+^ *Rosa26*^invHC/+^ (red) and *Rosa26*^invHC/+^ (gray) mice. The flow-cytometric definition of the different cell types is described in Materials and methods. DN T, CD4^−^CD8^−^ double-negative thymocytes; DP T, CD4^+^CD8^+^ double-positive thymocytes; Ery, erythrocytes; Gran, granulocytes. **(H)** Immunofluorescent staining of coronal brain sections of an adult control *Pax5*^+/+^ *Rosa26*^invHC/+^ brain. Left: Absence of mCherry expression (red) in the presence of DAPI staining (blue). Right: Mirror image showing the absence of mCherry expression (gray) without DAPI staining. The scale bar denotes 2 mm. The two sections were taken from the brain locations indicated by their reference to the Bregma landmark shown in the top right corner. CB, cerebellum; CH, cerebrum; IB, interbrain; MB, midbrain; MY, medulla oblongata of myelencephalon. Unpaired *t* test (C and D); *, P < 0.05. For detailed statistical information (C and D), see Table S6. Each dot (C and D) represents one mouse.

During embryonic development, Pax5 is expressed at the isthmic organizer, where it orchestrates midbrain and cerebellum development in cooperation with Pax2 (Schwarz et al., 1997). However, the precise role of Pax5 in cerebellar development is unknown. Given the aberrant cerebellar foliation in *Pax5*^R31Q/−^ and *Pax5*^−/−^ mice (Urbánek et al., 1994; Fig. 8, B and C) and the lack of *Pax5* expression in the adult cerebellum (Stoykova and Gruss, 1994), we hypothesized that *Pax5* is expressed in cerebellar progenitor cells during development. To test this hypothesis, we generated a *Pax5*^Cre^ allele by inserting a Cre gene into the second exon of the *Pax5* locus (Fig. S5, A–D) to trace the developmental trajectory of the cerebellum to *Pax5*-expressing progenitor cells. For this, we also generated a novel Cre-dependent mCherry reporter line by inserting a H2B-mCherry (HC) fusion gene in inverted orientation between convergent *lox* sites into the *Rosa26* locus (*Rosa26*^invHC^; Fig. S5, E and F). We validated this new reporter system in *Pax5*^Cre/+^ *Rosa26*^invHC/+^ mice by demonstrating that only Pax5-expressing B-lineage cells within the hematopoietic system gave rise to HC expression upon Cre-mediated inversion of the reporter cassette (Fig. S5 G).

**Figure 10.**
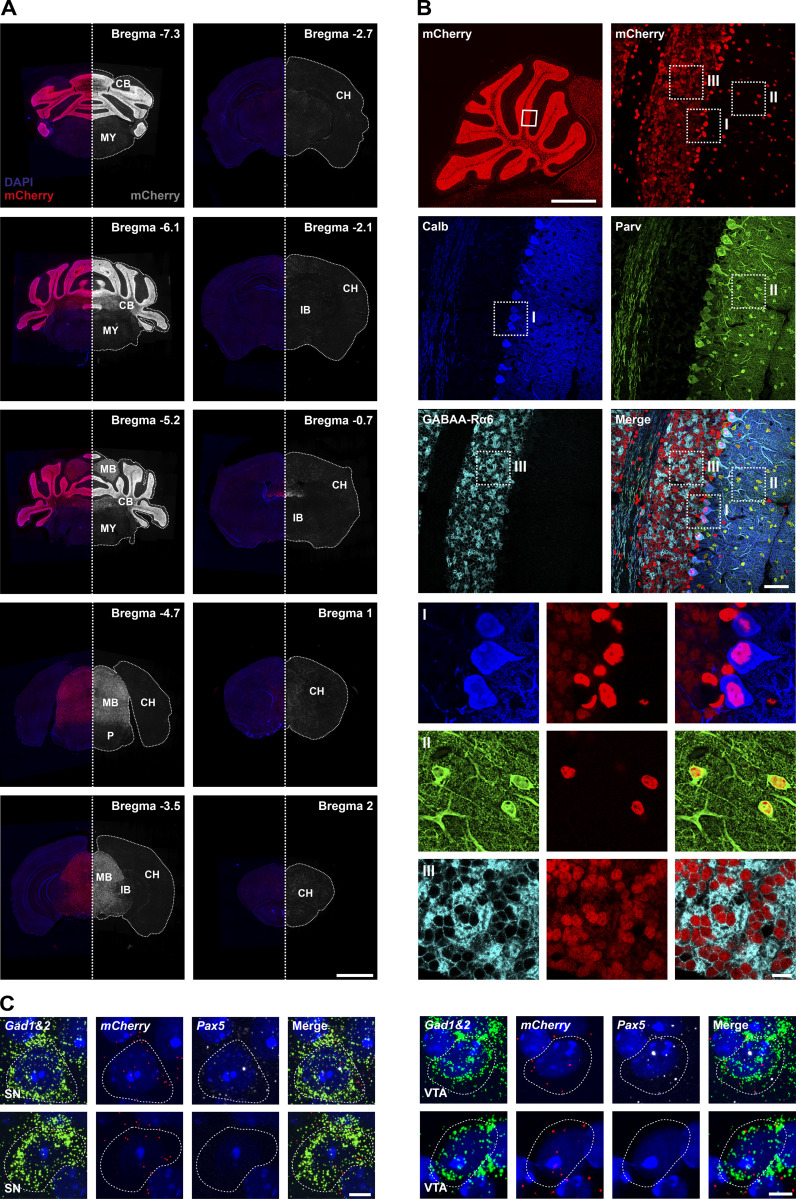
***Pax5*-expressing progenitors predominantly contribute to midbrain and cerebellar development. (A)** HC expression in an adult *Pax5*^Cre/+^ *Rosa26*^invHC/+^ brain, analyzed by immunofluorescence staining of coronal sections sampled at regular intervals along the posterior–anterior axis of the brain. Left: mCherry expression (red) with DAPI staining (blue). Right: A mirror image showing mCherry expression in gray. White dotted lines outline the circumference of the sections. The different sections were taken from the brain locations indicated by their reference to the Bregma landmark shown in the top right corner. The scale bar denotes 2 mm. CB, cerebellum; CH, cerebrum; IB, interbrain; MB, midbrain; MY, medulla oblongata of myelencephalon; P, pons. **(B)** Lineage-tracing analysis of cerebellar cell types. Top: Sagittal section of an adult *Pax5*^Cre/+^ *Rosa26*^invHC/+^ brain indicating HC reporter protein expression (red) in the entire cerebellum (left) or indicated insert (right). Middle: Anti-calbindin (blue), anti-parvalbumin (green), or anti–GABAA-R6α (cyan) staining is shown together with a merged image. The indicated inserts contain Purkinje cells (I), interneurons (II), or granule cells (III). Bottom: Individual antibody staining and mCherry expression together with a merged image of insets I–III. Scale bars: 1 mm (overview); 50 μm (middle); 10 μm (I–III). **(C)** smRNA-FISH analysis of the SN and VTA midbrain regions on coronal sections of an adult *Pax5*^Cre/+^ *Rosa26*^invHC/+^ brain, analyzed with probes for *Gad1*/*Gad2* (green), *mCherry* (red), and *Pax5* (white) in combination with DAPI (blue). The scale bar denotes 5 μm. The data (A–C) are representative of three independent experiments.

Immunofluorescent analysis of mCherry expression in the adult brain of *Pax5*^Cre/+^ *Rosa26*^invHC/+^ mice showed ubiquitous expression of the reporter protein in the cerebellum and midbrain, but also some scattered expression in the forebrain, pons, and medulla oblongata (Fig. 10 A). Within the cerebellum, mCherry was expressed in the majority of Purkinje cells, granule cells, and molecular layer interneurons, thereby establishing that the cerebellum originates from *Pax5*-expressing progenitor cells (Fig. 10 B). Importantly, mCherry was not expressed in the midbrain and cerebellum of control *Pax5*^+/+^ *Rosa26*^invHC/+^ mice (Fig. S5 H).

We next investigated whether GABAergic midbrain neurons originate from *Pax5*-expressing progenitor cells. smRNA FISH analysis revealed concomitant expression of *mCherry* and *Gad1*, *Gad2* mRNA in the VTA and SN in cells with and without active *Pax5* expression (Fig. 10 C), demonstrating that GABAergic neurons in these regions without active *Pax5* expression also originate from *Pax5*-expressing progenitor cells. In summary, these lineage-tracing experiments demonstrated a critical role of Pax5 in the neurogenesis of GABAergic midbrain neurons.

## Discussion

*PAX5* is well known for its role in B cell immunity (Calderón et al., 2021; Horcher et al., 2001; Nutt et al., 1999) and its function as a haploinsufficient tumor suppressor gene in B cell acute lymphoblastic leukemia (Gu et al., 2019; Mullighan et al., 2007). Here, we identified new functions of Pax5 in controlling cerebellar development and the neurogenesis of GABAergic midbrain neurons and demonstrated a causative involvement of *PAX5* deficiency in ASD etiology. Hence, both the B cell– and brain-specific functions of Pax5 are each associated with a pathological condition caused by partial loss of PAX5. Notably, the *Pax5*^R31Q/−^ mouse model largely recapitulated the immunological and neurodevelopmental deficits of the patient, thus demonstrating that the biallelic *PAX5* mutations of the patient are sufficient to explain these phenotypes. Whereas B cell development was normal in *Pax5*^+/−^ mice, it was partially arrested at the pro-B cell stage and led to greatly impaired B cell immune responses in *Pax5*^R31Q/−^ mice, thus recapitulating the immunological phenotype of the patient. Hence, biallelic *PAX5* mutations cause a novel form of hypogammaglobulinemia. At the molecular level, the hypomorphic R31Q mutation selectively interfered with DNA binding of Pax5 in *Pax5*^R31Q/−^ pro-B cells, resulting in the deregulation of 10 and 20% of the known Pax5-repressed and Pax5-activated genes, respectively. These gene expression changes were still compatible with survival of the *Pax5*^R31Q/−^ mice to adulthood, in contrast to the early death of *Pax5*^−/−^ mice at weaning age (Urbánek et al., 1994). The absence of a haploinsufficient effect on B cell development contrasts with the neurodevelopmental phenotypes observed in *Pax5*^+/−^ mice, which may be explained by the continuous regeneration of B cells from hematopoietic stem cells throughout life in contrast to the postmitotic neural tissue.

While previous genetic studies have suggested *PAX5* haploinsufficiency as a risk factor for ASD (Gofin et al., 2022; Iossifov et al., 2012; O’Roak et al., 2014; Stessman et al., 2017), our results provide causal evidence for the role of *PAX5* in the etiology of ASD. The social phenotype of known ASD mouse models can range from asocial behavior (Tsai et al., 2012; Won et al., 2012) to hypersociability (Fountain et al., 2017; Katayama et al., 2016; Stoppel and Anderson, 2017). Notably, the *Pax5*^R31Q/−^ mouse exhibited abnormal social behavior, which manifested as hypersociability that could reflect the social disinhibition observed for the patient. It should, however, be noted that, in mice, excessive sniffing behavior and an orientation towards new social stimuli may also be associated with hyperactivity. Furthermore, human social behavior is far more complex than social behavior in rodents, and consequently, mice mirror only a small fraction of the behavioral abnormalities observed in ASD. The hypersociability trait can be explained by the depletion of GABAergic neurons in the VTA of *Pax5*^R31Q/−^ mice, given that the VTA mediates social reward (Hung et al., 2017) and that VTA excitability controls the preference for nonfamiliar mice (Bariselli et al., 2018). Furthermore, aberrant cerebellar foliation could also contribute to this phenotype, given that the cerebellum and VTA are functionally connected and that this pathway modulates social behavior (Carta et al., 2019). *Pax5*^R31Q/−^ mice buried more marbles, which is a phenotype associated with repetitive behavior (Thomas et al., 2009), a core ASD feature. Unexpectedly, these mice spent less time self-grooming. This observation could be explained by the aberrant anatomy of cerebellar lobule VII in *Pax5*^R31Q/−^ mice, consistent with a previous study showing less self-grooming upon developmental perturbation of lobule VII (Badura et al., 2018). Furthermore, both the patient as well as the *Pax5*^R31Q/−^ mouse showed structural cerebellar abnormalities, which are a common pathological finding in ASD (Wang et al., 2014) and are recapitulated in many mouse models (Badura et al., 2018).

The profound loss of the GABAergic innervation seen in the VTA and SN of *Pax5*^R31Q/−^ mice suggests that the balance between excitatory (E) and inhibitory (I) circuits is altered in the midbrain. An appropriate E/I ratio is critical for regulating brain activity and information processing (Nelson and Valakh, 2015). Disruption of the E/I balance has become a dominant theory concerning the pathogenesis of ASD and other neurodevelopmental disorders (Nelson and Valakh, 2015; Rubenstein and Merzenich, 2003; Uzunova et al., 2016). While most studies have focused on the E/I balance in the neocortex and hippocampus, our data imply that the inhibitory circuitry of the midbrain also needs to be addressed in future studies.

Furthermore, *Pax5*^R31Q/−^ mice showed sensorimotor deficits, hyperactivity, and learning delays, consistent with the motor control deficits, restlessness, and cognitive impairment observed in the patient. Sensorimotor deficits are common in ASD (Bath, 2020) and often result from dysfunction of the cerebellum (Gowen and Miall, 2007). The failure of the patient to perform the anti-saccade and memory-saccade tasks is consistent with the observed abnormal cerebellar anatomy, given that the cerebellum is essential for eye movement control (Pretegiani et al., 2018). Hyperactivity was consistently seen across several behavioral assays, in which *Pax5*^R31Q/−^ mice covered more distance compared with their wild-type littermates and displayed shorter burying and grooming bouts. These behavioral deficits may arise as a consequence of the anatomic abnormalities in the cerebellum, VTA, or SN. Given that chronic overactivation of the VTA leads to hyperactivity (Boekhoudt et al., 2017), we propose that the dramatic loss of GABAergic neurons in the VTA of *Pax5*^R31Q/−^ mice may also be linked to hyperactivity in addition to hypersociability. Similarly, the loss of GABAergic innervation in the SN is also likely to contribute to the hyperactivity observed in *Pax5*^R31Q/−^ mice, considering that, in humans, increased activity of the SN is associated with motor impulsivity (Zhang et al., 2017) and that direct stimulation of the SN elicits movement (Barter et al., 2015). Notably, *Pax5*^+/−^ mice exhibited abnormal social and repetitive behaviors, coordination deficits, hyperactivity, and sensorimotor performance and learning deficits, albeit often in a milder form, indicating that *PAX5* haploinsufficiency is already sufficient to cause a neurodevelopmental disorder.

The abundant expression of Pax5 in the isthmic organizer at the midbrain–hindbrain boundary of the embryo (Urbánek et al., 1994) is in stark contrast to its sparse expression in the adult brain (this study). Lineage tracing revealed that Pax5-expressing progenitors prominently contribute to adult midbrain and cerebellar development. These results support an important developmental role of Pax5 in midbrain and cerebellar morphogenesis, including the differentiation of midbrain GABAergic neurons, and thus provide a mechanistic basis for understanding why the loss of Pax5 leads to neurodevelopmental abnormalities in mouse and humans.

## Materials and methods

### Patient

The family of the patient provided informed consent to WES, to use their samples for research, and to publish the acquired data in accordance with the Helsinki principles for the enrolment in research protocols, which were approved by the Institutional Review Boards of Erasmus University Medical Center (Ethical Commission number MEC-2013-026). Blood from healthy donors was obtained based on approved protocols (MEC-2021-0251). All relevant ethical requirements for working with human study participants were complied with.

### Patient description

#### Clinical description of the PAX5 mutant patient

The patient, a Caucasian man of a non-consanguineous parents, was born after 40 wk of gestation with APGAR scores of 9 and 10, a birth weight of ∼3,600 g, and an uneventful initial postpartum period. Neither parent has any health issues. The family also has one healthy daughter who has an above-average educational level.

#### Immunological assessment (by V.A.S.H. Dalm)

The patient presented with recurrent oral thrush in the first 3 mo of life. From the age of 8 mo onwards, he suffered from recurrent upper respiratory tract infections, including sinusitis and otitis, for which he was treated with antibiotics, and underwent surgery for adenoidectomy and ear tube insertion. Recurrent infections were associated with failure to thrive. At the age of 2.5 yr, he developed pneumococcal pneumonia and meningitis. There was an uneventful recovery after i.v. antibiotic treatment. During admission, it was found that serum immunoglobulins were nearly absent, thereby fulfilling the diagnostic criteria for hypogammaglobulinemia (IgG < 1.7 g/liter [reference value 3.5–10 g/liter]; IgA < 0.067 g/l [0.19–1.1 g/liter]; and IgM < 0.3 g/l [0.3–1.4 g/liter]). Further immunological workup at that time showed a reduction in the naive, natural effector, and memory B cell subsets, but normal transitional B cell numbers and no abnormalities in the T cell compartment (Table S1). Genetic analysis did not show variants in *BTK*. He commenced on immunoglobulin replacement therapy (IGRT) because of hypogammaglobulinemia with recurrent and severe bacterial infections at the age of 2.5 yr. After initiation of the therapy, there was a significant clinical improvement, with decline in number and severity of infections, and the patient started to gain weight again, which gradually developed into obesity (current BMI of 30.86 kg/m^2^). He did not suffer from any infections until the age of 12 yr, but then developed recurrent upper respiratory tract infections. His trough level of IgG was 5.8 g/liter at the time, and the dose of IGRT was increased. He did not suffer from infections necessitating antibiotic treatment and/or hospitalization after this period. WES did not reveal pathogenic or candidate variants in primary immunodeficiency–associated genes but biallelic mutations in the *PAX5* gene. At the age of 14, a chest computed tomography scan revealed bronchiectasis in the right middle lobe, for which antibiotic prophylaxis was started (500 mg azithromycin three times per week). To date, he does not suffer from infectious complications affecting other organ systems, including the skin and gastrointestinal tract. There are no signs of autoimmune, autoinflammatory, or lymphoproliferative complications or solid or hematological malignancies.

#### Neurodevelopmental observations

The patient initially showed delayed development. Development of gross and fine motor skills and speech language were slightly delayed. The patient began walking at the age of 18 mo and began speaking after 12 mo, and at the age of 3 yr, he was capable of saying short sentences. After the start of IGRT, his delayed psychomotor development became more apparent. He had sleeping difficulties during the first years of his life. At kindergarten age, his parents thought that he was impulsive and clumsy, and he had few friends. At school age, he had physical therapy and speech-language therapy. Puberty started when he was 10 yr of age. His IQ was regularly tested from the age of 4 yr onwards, and his scores ranged between 59 and 80, although most assessments reported a mild intellectual disability. He attended special education for children with learning disabilities, and to date he participates in a work/day-care project. He was diagnosed with an ASD at the age of 5 yr.

#### Instruments used for neuropsychological assessment

##### ADOS-2, module 4

The Autism Diagnostic Observation Schedule, second version (ADOS-2; Norms Dutch adaptation 2013) is an instrument that identifies various symptoms of an ASD through observation and semistructured interviews (Table S2). Higher scores indicate more ASD-related symptoms.

##### BRIEF

The Behavior Rating Inventory of Executive Function has several scales that together give an impression of the executive functioning of a person. Executive functions are cognitive processes necessary for goal-directed, efficient, and social behavior. Since both scales are normed for adolescents up to 17 yr old, we used norms of the oldest age category.

##### CBCL 6-18 and YSR

The Child Behavior Checklist is a parent-report tool used to screen for behavioral and emotional problems. The Youth Self Report is a self-rating scale with comparable content. Since both scales are normed for adolescents up to 18 yr old, we used norms of the oldest age category.

##### DCD-Q

The Developmental Coordination Disorder Questionnaire is a parent-report identification tool used to screen children for the presence of motor impairments. Lower scores indicate more problems. Since this scale is normed for ages 7–14, we used norms of the oldest age category.

##### SCL-90-R

The Symptom Check List 90-Revised Dutch version 2004 (SCL-90-R) is a self-report questionnaire of multidimensional complaints. Higher scores indicate more problems.

##### SRS-2

The Social Responsiveness Scale-2 identifies social impairment associated with ASD and quantifies the severity. It detects subtle symptoms, and differentiates clinical groups, both within the ASD. Since this scale is normalized for adolescents up to 18 yr old, we used norms of the oldest age category.

##### WAIS-IV-NL

Dutch version of the fourth revision of the Wechsler Adult Intelligence Scales (Norms 2012, Dutch/Flemish norm group 18; 0–19; 11 yr).

#### Neuropsychological assessment (by A. Rietman)

At the age of 19 yr, the patient had a neuropsychological assessment at the Department of Child and Adolescent Psychiatry/Psychology of the Erasmus MC by a licensed neuropsychologist (A. Rietman) to get an impression of the strengths and weaknesses in his cognitive and behavioral profile. At that time, the patient was described as a cooperative adolescent whose cognitive skills were below average (WAIS-IV-NL; Table S2). Within his cognitive profile, most scores were in the below-average range, except for the subtest on motor coordination and speed (Digit Symbol Coding). However, his scores were higher compared with an assessment performed 2 yr earlier. Although the scores are below average, they are no longer within the intellectual disability range.

On the ADOS-2 observation scales for adolescents and adults, the patient meets the criteria for an ASD. During the assessment, we noticed that the patient was somewhat restless. We evaluated symptoms of attention-deficit/hyperactivity disorder (ADHD) in a structured interview with him and his mother. There appeared to be mainly physical restlessness, but most other criteria for ADHD were not met. On the ADOS-2 observation scales for adolescents and adults, the patient meets the criteria for an ASD (Table S2).

On the SCL 90-R self-report questionnaire of complaints, the patient mainly shows complaints in the areas of agoraphobia (anxiety in open spaces), hostility, and sleeplessness. Although the patient himself does not recognize any problems in the area of executive functions, his mother indicates problems in scales for behavioral regulation (inhibition and shifting) and metacognition (working memory and monitoring) on the BRIEF scales for problems in executive functioning (Table S2).

The patient’s mother completed a questionnaire on motor proficiency, the DCD-Q. These scores indicated that a developmental coordination disorder (DCD) assessment and diagnosis should be considered. The rating scales for behavioral and emotional problems (CBCL and YSR) mainly indicated internalizing problems. Scores from the mother and the patient himself showed a high degree of agreement. The mother scored the patient in the clinical range on the scales for somatic problems, thought problems, attention problems, and rule-breaking behavior. Patient scored himself in the clinical range on the scales for social problems, thought problems, and attention problems (Table S2).

Finally, scores on the autism screening scale SRS-2 showed total scores in the clinical range compared with the general population. The patient’s mother indicated high scores on scales for social communication, social motivation, restricted interests, and repetitive behavior. Both scores on the DSM-oriented scales (social communication and interaction as well as restricted interests and repetitive behavior) were in the clinical range (Table S2). The patient has no close friends, and he does not engage in social activities on his own accord. He enjoys being alone and has a low social motivation. He does, however, incidentally ask a lot of questions during social interactions, and his questions are somewhat inappropriate. For example, he asked two researchers, “Are you married to each other?” While the question can be seen as interested, this tendency can also be regarded as socially disinhibited.

To conclude, the patient is a 19-yr-old man with below-average intellectual capabilities. The diagnosis of an ASD can be confirmed. In addition to these communicative and social problems, there are motor, emotional, behavioral, attention, and sleeping problems and restlessness.

### Mice

The following mice were maintained on the C57BL/6 genetic background: *Pax5*^+/−^ (Urbánek et al., 1994), *Pax5*^fl/fl^ (Horcher et al., 2001), *Rag2*^−/−^ (Shinkai et al., 1992), *Igh*^∆Jh/∆Jh^ (J_H_T; Gu et al., 1993), and transgenic *Vav-*Cre (de Boer et al., 2003). All animal experiments were carried out at the Research Institute of Molecular Pathology (Vienna) according to valid project licenses, which were approved and regularly controlled by the Austrian Veterinary Authorities. All animal experiments performed at the Erasmus MC (Rotterdam) were approved by an independent animal ethics committee (DEC-Consult) and conformed to the relevant institutional regulations of the Erasmus MC and Dutch legislation on animal experimentation (CCD approval: AVD101002015273 and AVD1010020197846).

### Generation of mutant *Pax5* alleles

The *Pax5*^R31Q^ and *Pax5*^E242^* alleles were generated by CRISPR/Cas9-mediated genome editing in mouse zygotes (Yang et al., 2013; Fig. S1, A and B). For this, mouse zygotes (C57BL/6 × CBA) were injected with Cas9 mRNA, an sgRNA targeting the sequence to be mutated (linked to the scaffold tracrRNA), and a single-stranded DNA repair template of 200 nucleotides (Table S7) to introduce the specific mutation at *Pax5* codon 31 or 242 to generate the *Pax5*^R31Q^ and *Pax5*^E242^* alleles (Fig. S1, A and B), respectively. Mice carrying the introduced mutation were identified by PCR amplification of the respective genomic DNA fragment (Table S7) and subsequent restriction digestion with HhaI (R31Q) and XbaI (E242*) followed by verification of the mutations by DNA sequencing of the respective PCR fragment (Fig. S1, A and B). The mutant *Pax5* alleles were backcrossed to the C57BL/6 background before analysis.

The *Pax5*^Cre^ allele was generated by in-frame insertion of a codon-improved (i) Cre gene after the ninth codon of *Pax5* exon 2 (Fig. S5 A) flanked by six copies of a synthetic poly(A) site, which was designed based on the rabbit β-globin poly(A) sequence (Levitt et al., 1989). A building vector containing this Cre gene insertion flanked by 250-bp homology regions corresponding to the 3′ end of *Pax5* intron 1 and the 5′ end of intron 2, respectively, was used to generate single-stranded DNA, which was injected with Cas9 protein and *Pax5* exon 2–specific sgRNAs (Table S7) into mouse zygotes (C57BL6 × CBA) according to the *Easi*-CRISPR method (Miura et al., 2018; Fig. S5 A). The *Pax5*^Cre^ allele was subsequently backcrossed to the C57BL/6 background. Primers 1–3, shown in Table S7, were used for PCR genotyping of the *Pax5*^Cre/+^ mice. The *Pax5*^Cre^ allele was identified by amplifying a 353-bp PCR fragment with primer pair 1/2, and the wild-type *Pax5* allele, by amplifying a 558-bp PCR fragment with primer pair 1/3 (Fig. S5 B).

### Generation of the *Rosa26*^invHC^ allele

DNA sequences encoding the human histone H2B fused in frame to mCherry (HC) were inserted in inverted (inv) orientation between convergent *lox*66 and *lox*71 sites (Anastassiadis et al., 2010) downstream of a CMV enhancer and chicken actin promoter into a *Rosa26* targeting vector containing a *frt*-flanked *Pgk1* promoter-neomycin resistance (Neo^r^) gene cassette (Fig. S5 E). The linearized targeting vector was electroporated into HM-1 ES cells (Magin et al., 1992) followed by neomycin selection, identification of PCR-positive clones, and subsequent removal of the neomycin selection cassette by electroporation with a Flpe recombinase–expressing vector. Correctly targeted ES cell clones were verified by Southern blot analysis before injection into C57BL/6 blastocysts and the generation of *Rosa26*^invHC/+^ mice. The *Rosa26*^invHC^ allele was subsequently backcrossed to the C57BL/6 background. The *Rosa26*^HC^ allele containing the HC gene in the correct transcriptional orientation was generated by Cre-mediated reversal of the inverted HC insert of the *Rosa26*^invHC^ allele (Fig. S5 E). Primers 1–4, show in Table S7, were used for PCR genotyping of the three *Rosa26* alleles. The *Rosa26*^invHC^ allele was identified by amplifying a 552-bp PCR fragment with primer pair 1/3; *Rosa26*^HC^ allele, by amplifying a 465-bp PCR fragment with primer pair 2/3; and the wild-type *Rosa26* allele, by amplifying a 253-bp PCR fragment with primer pair 4/3 (Fig. S5, E and F).

### Antibodies

The following monoclonal antibodies were used for flow-cytometric analysis of mouse lymphoid organs from 3–12-wk-old mice: B220/CD45R (RA3-6B2), CD2 (RM2-5), CD3 (17A2), CD4 (GK1.5), CD5 (53-7.3), CD8a (53-67), CD11b/Mac1 (M1/70), CD19 (6D5) or CD19 (1D3), CD21/CD35 (7G6), CD23 (B3B4), CD49b (HMa2), CD93 (AA4.1), CD95/Fas (Jo2), CD117/Kit (ACK2), CD127/IL7-Rα (A7R34), CD135/Flt3 (A2F10.1), CD138 (281-2), CD267/TACI (8F10), GL7 (GL-7), Gr1 (RB6-8C5), IgD (11-26C), IgE (R35-72), IgG1 (A85-1), IgM (II/41) or IgM (eb121-15F9), Ly6C (HK1.4), Ly6D (49H4), NK1.1 (PK136), Sca1 (D7), TCRβ (H57-597), and Ter119 (Ter-119) antibodies. The following antibodies were used for flow-cytometric analysis of human blood: CD3 (SK7), CD24 (ML5), CD27 (M-T271), CD38 (HIT2), IgA (IS11-8E10), IgD (IA6-2), IgG (G18-145), and IgM (MHM-88) antibodies.

The anti-Pax5 antibody (directed against amino acids 17–145; Adams et al., 1992) was used for ChIP and immunoblot analysis. The following antibodies were used for immunoblot analysis: anti-Pax5 (HPA056394; Sigma-Aldrich), anti-GAPDH (14C10 or D16H11; Cell Signaling Technology), anti-H3 (96C10; Cell Signaling Technology), and anti–β-actin (ACTB; AC-15; Abcam). The following antibodies were used for intracellular staining and phospho-specific flow cytometry: IgM (eb121-15F9), Pax5 (1H9; BD Bioscience), phospho-AKT (p-Ser473; D9E; Cell Signaling Technology), and PTEN (138G6; Cell Signaling Technology).

The following antibodies were used for immunohistochemical analysis: anti-GABAA receptor 6α (G5544; Sigma-Aldrich), anti-calbindin (214006; Synaptic Systems), anti-parvalbumin (McAB235; Swant), anti-tyrosine hydroxylase (ab76442; Abcam), anti-aldolase C (mouse antibody; gift from R. Hawkes’ laboratory, Department of Cell Biology & Anatomy and Hotchkiss Brain Institute, Faculty of Medicine, University of Calgary, Calgary, Canada), and anti-Pax5 (HPA056394; Sigma-Aldrich).

### Definition of cell types by flow cytometry

The different hematopoietic cell types of the mouse were identified by flow cytometry as follows: multipotent progenitor (Lin^–^ CD135^+^ Kit^hi^ Sca1^hi^), all-lymphoid progenitor (Lin^−^ CD135^+^ CD127^+^ Ly6D^−^), B cell–biased lymphoid progenitor (Lin^–^ CD135^+^ CD127^+^ Ly6D^+^), pro-B (B220^+^ CD19^+^ Kit^+^ CD2^−^ IgM^−^ IgD^−^), pre-B (B220^+^ CD19^+^ Kit^−^ CD2^+^ IgM^−^ IgD^−^), immature B (B220^+^ CD19^+^ IgM^hi^ IgD^−^), recirculating B (B220^+^ CD19^+^ IgM^+^ IgD^hi^), MZ B (B220^+^ CD19^+^ CD93^−^ CD21^hi^ CD23^lo/−^), FO B (B220^+^ CD19^+^ CD93^−^ CD21^int^ CD23^hi^), GC B (B220^+^ CD19^+^ Fas^+^ GL7^+^), plasma (CD138^hi^ TACI^hi^), NP^+^ plasma (NP_29_-Phycoerythrin^+^NP_14_-CGG-Alexa-Fluor-488^+^ CD138^hi^ TACI^hi^), B-1a (B220^lo^ CD19^+^ CD23^−^ CD5^+^), B-2 (B220^+^ CD19^+^ CD23^+^ CD5^−^), total B (B220^+^ CD19^+^), and total T (CD3^+^ or TCRβ^+^) cells; double-negative (DN) thymocytes (CD4^−^ CD8^−^ Thy1.2^+^), double-positive (DP) thymocytes (CD4^+^ CD8^+^), granulocytes (Gr1^hi^ Mac1^hi^), and erythrocytes (Ter119^lo^ Kit^−^). Multipotent progenitors, all-lymphoid progenitors, and B cell–biased lymphoid progenitors were defined by electronically gating away Lin^+^ cells with a cocktail of anti-CD3, CD4, CD8, CD11b, CD19, CD49b, Gr1, Ly6C, NK1.1, and Ter-119 antibodies. Human T and B cells were defined as follows: T (CD3^+^), B (CD19^+^), naive mature B (CD19^+^ CD27^−^ IgD^+^ CD38^−^ CD24^−^), transitional B (CD19^+^ CD27^−^ IgD^+^ CD38^+^ CD24^dim^), and natural effector B (CD19^+^ CD27^+^ IgD^+^) cells. Flow cytometry experiments and FACS sorting were performed on LSRFortessa (BD Biosciences) and FACSAria III (BD Biosciences) machines, respectively. FlowJo Software (TreeStar) was used for data analysis.

### RT-qPCR analysis of *PAX5* mRNA expression

Naive mature B cells, which were sorted from peripheral blood mononuclear cells of the patient, were used for total RNA preparation with the RNeasy Plus Kit (Qiagen). cDNA was synthesized using the High-Capacity cDNA Reverse Transcription Kit (Applied Biosystems). *PAX5* full-length transcripts were amplified by PCR using the Platinum Taq DNA Polymerase High Fidelity kit (Invitrogen) with the primers shown in Table S7 and were cloned into the pcDNA3.1(+) expression vector. Plasmid DNA of single clones was prepared, followed by Sanger sequencing of the cloned *PAX5* cDNA sequences.

### Transient transfection and luciferase assays

Human *PAX5* cDNA cloned in the pcDNA3.1(+) expression vector was used to generated the PAX5-R31Q and PAX5-E242* mutants by site-directed mutagenesis using the QuikChange XL Site-Directed Mutagenesis Kit (Agilent). The PAX5 expression plasmids were transfected together with the lucCD19 (Czerny and Busslinger, 1995) and pRL-CMV constructs (Promega) into HEK293T cells (ATCC), using the FuGENE 6 Transfection Reagent (Promega). 2 d after transfection, luciferase activity was determined using the Dual-Glo Luciferase Assay System (Promega) on a GloMax Discover Microplate Reader (Promega). The ratio of the luminescence of the experimental firefly luciferase reporter to the luminescence of the control renilla luciferase reporter was calculated and normalized to the vector control.

### Intracellular staining and phospho-specific flow cytometry

Intracellular Pax5 staining of pro-B cells (Fig. 2 D) and intracellular Igμ staining of pro- and pre-B cells (Fig. S1 C) were performed after fixation-permeabilization with the Foxp3 Staining Buffer Set (eBioscience). Analysis of PTEN and phosphorylated AKT (Ser 473) levels (Fig. 4, A and B) was performed with lymph node CD43^−^ FO B cells, which were incubated for 1 h at 37°C in RPMI-1640 containing 10% heat-inactivated FCS, 2 mM glutamine, and 50 μM β-mercaptoethanol followed by a 30-min stimulation with 10 μg/ml goat anti-mouse IgM F(ab′)_2_ fragment (Jackson ImmunoResearch Laboratories), as described (Calderón et al., 2021). Stimulated and nonstimulated cells were fixed, permeabilized, and stained with antibodies detecting CD19, phospho-AKT (p-Ser 473), or PTEN for 1 h at RT, followed by flow-cytometric analysis.

Human peripheral blood mononuclear cells were resuspended in RPMI-1640 supplemented with 5% FCS, 25 mM Hepes, 2 mM glutamine, and 55 μM β-mercaptoethanol at a density of 1 × 10^6^ in 0.1 ml, stimulated with 20 μg/ml goat anti-human IgM-UNLB F(ab′)_2_ fragment (SouthernBiotech) for 30 min at 37°C, fixed, and stained with an anti–phospho-AKT (p-Ser473) antibody and additional cell surface antibodies to identify naive mature B cells by flow-cytometric analysis.

### Immunization and ELISA analysis

Immunization with a T cell–independent antigen was performed by i.p. injection of mice with 50 μg NP-Ficoll (Fig. 4, C and D). The immune response to a T cell–dependent antigen was analyzed by i.p. injection of mice with 100 μg NP-KLH in alum (Fig. 4, E–G). The serum titers of NP-specific IgM, IgG, and IgG1 antibodies were determined by ELISA using plates that were coated with 25 μg/ml NP_24_-BSA or NP_7_-BSA to capture total or high-affinity NP-specific antibodies, respectively (Fig. 4 G). The serum concentration of NP-specific IgG1 was determined relative to that of a standard NP-specific IgG1 antibody (hybridoma SSX2.1). The titers of total IgM, IgG, and IgA in the serum of nonimmunized mice (Fig. 3 F) were measured by ELISA, using plates coated with 1 μg/ml of anti-IgM, anti-IgG, or anti-IgA antibodies (Southern Biotechnology Associates), and were calculated relative to purified IgM, IgG, or IgA protein standards.

### Immunohistological analysis of the spleen

Spleen sections from NP-KLH–immunized mice (Fig. S1 H) were analyzed by immunofluorescence staining with APC-anti-CD3 (145-2C11; eBioscience), Alexa Fluor 488–anti-IgD (11-26c.2a; BioLegend), and biotinylated PNA (Vector Laboratories), which was detected with Cy3-Streptavidin (Jackson ImmunoResearch), as described (Calderón et al., 2021).

### ChIP-seq experiments

Pro-B cells were short-term cultured on OP9 cells in IL-7–containing IMDM (Nutt et al., 1997) followed by crosslinking with 1% formaldehyde (Sigma-Aldrich) for 10 min. Nuclei were prepared and lysed in the presence of 0.25% SDS, followed by sonication of the chromatin with the Bioruptor Standard (Diagenode). Immunoprecipitation was performed with an anti-Pax5 paired domain antibody (Adams et al., 1992), and the precipitated DNA (1–2 ng) was used for library preparation and subsequent Illumina deep sequencing (Table S8).

### cDNA preparation for RNA-seq

RNA from ex vivo–sorted pro-B cells was isolated with a RNeasy Plus Mini kit (Qiagen), and mRNA was obtained by poly(A) selection with a Dynabeads mRNA purification kit (Invitrogen) followed cDNA synthesis as described (Calderón et al., 2021).

### Library preparation and Illumina deep sequencing

About 1–5 ng of cDNA or ChIP-precipitated DNA was used for generating sequencing libraries with the NEBNext Ultra Ligation Module and NEBNext End Repair/dA-tailing module, as described (Calderón et al., 2021). Cluster generation and sequencing were carried out by using the Illumina HiSeq 2000 system with a read length of 50 nucleotides (Table S8).

### Bioinformatic analysis of ChIP-seq data

#### Sequence alignment

All sequence reads of the different samples that passed the Illumina quality filtering were considered for alignment to the mouse genome assembly version of December 2011 (GRCm38/mm10) using the Bowtie 2 program v2.3.5.1 (Langmead and Salzberg, 2012). Read coverages (displayed in Figs. 5 and S2) were calculated with the BEDTool program v2.27.1 (Quinlan and Hall, 2010), normalized to reads per millions using SAMTools v1.9 (Li et al., 2009) and KentTools v20190507 (Kuhn et al., 2013), and visualized with the UCSC genome browser.

#### Peak calling

Where necessary (e.g., for comparisons of peak regions), reads were down-sampled to the lowest read number in the samples to be compared, using the Picard tool v2.18.27 (McKenna et al., 2010). Peaks were called with the magnetic-activated cell sorting (MACS) program v2.2.5 (Zhang et al., 2008), using only the reads of one DNA strand in the case of paired-end read samples, and a *Rag2*^−/−^ pro-B cell input sample (GSM1145867, GSM1296537) as a control. We filtered all peaks for P < 10^−10^ and assigned them to genes as described (Revilla-i-Domingo et al., 2012), based on the RefSeq database that was processed as described below. Peak overlap analyses were performed with the MULTOVL program (Aszódi, 2012). Read density profiles were generated with customized R scripts.

#### Motif discovery analysis

De novo prediction of sequence motifs was performed with the MEME program v5.0.4 (Bailey et al., 2015). For this, we extracted 300 nucleotides centered at the summit of the top-ranked 300 peaks among the common Pax5 peaks present in *Pax5*^+/+^ and *Pax5*^R31Q/−^ pro-B cells. Unique Pax5 peaks present only in *Pax5*^+/+^ pro-B cells were selected by filtering for the most different peaks between *Pax5*^+/+^ and *Pax5*^R31Q/−^ pro-B cells, as measured by normalized read count (counts per million [CPM]) differences. For this, we calculated the *Pax5*^R31Q/−^/*Pax5*^+/+^ difference of square root–scaled CPM values by applying sqrt(cpm_r31q/mean[cpm_r31qt])—sqrt(cpm_wt/mean[cpm_wt]).

### Bioinformatic analysis of RNA-seq data

#### Sequence alignment

For each sample, the reads of three sequencing runs were concatenated. Sequence reads that passed the Illumina quality filtering were filtered against rDNA with Bowtie 2 (Langmead and Salzberg, 2012). The remaining reads were aligned with the STAR program v2.4.2 (Dobin et al., 2013) to the mouse genome version of December 2011 (GRCm38/mm10). Uniquely mapping reads were used for gene expression analysis.

#### Gene annotation

Sequence alignment and database generation of the RefSeq-annotated genes was performed as previously described (Wöhner et al., 2016). In addition, the immunoglobulin λ light-chain segments were replaced with their corresponding converted GRCm38.p3 annotations (Yates et al., 2016), which resulted in a gene number of 25,579.

#### Differential gene expression

The number of reads per gene was counted using the featureCounts program of the Rsubread package v1.34.6 (Liao et al., 2013). The datasets were grouped according to genotype and were analyzed using the R package DESeq2 v1.24.0 (Love et al., 2014). Genes with low expression (CPM < 1 in all samples) were removed from the analysis. The normalizations and dispersion estimations of the samples were conducted using the default DESeq2 settings. Variance-stabilizing transformations were computed with the blind option set to False. Variance-stabilized counts were transformed from the log_2_ to the log_10_ scale for generating scatterplots (Fig. 5 A and Fig. S2, A and B). The default DESeq2 pairwise setup (model design formula ∼genotype; Wald test) was used for comparison between conditions. Genes with an adjusted P value of <0.05, an absolute fold-change of >3, and a mean transcripts per million (TPM) value (averaged within conditions) of >5 were called as significantly expressed. Immunoglobulin genes were filtered from the list of significantly expressed genes.

### Correlation of gene activation with differential Pax5 binding at the TSS

Peak-to-gene assignment was used to identify all Pax5 peaks that were located at the TSS region of genes. Pax5 binding was calculated as CPMs over the union region of these peaks at the TSS. The log_2_ ratio of the Pax5-binding difference at each TSS between *Pax5*^R31Q/−^ and *Pax5*^+/+^ pro-B cells was determined, and the cumulative log_2_ ratios were plotted for differentially activated genes (more than twofold), which were ranked according to their gene expression difference (Fig. S2 F). As a control, the ranking of the activated genes was 100 times randomly shuffled to generate the randomized data of the binding differences shown in Fig. S2 F. The median Pax5-binding difference at the TSS regions was determined for all activated genes (more than twofold between *Pax5*^R31Q/−^ and *Pax5*^+/+^ pro-B cells) as well as for nonregulated genes with an expression of >5 TPM (Fig. 5 F). The *R* program v3.6.0 (https://www.r-project.org) was used for all calculations and plotting of the data.

### Human behavioral tasks

The experimental design of the visuomotor adaptation task was adapted from Jonker et al. (2020 *Preprint*) and performed as previously published (van der Vliet et al., 2018; see Video 1 and Fig. 6 A). The visuomotor assessment tasks were executed as previously described (Owens et al., 2018; Staal et al., 2021; see Video 2).

### Mouse behavioral experiments

The experimenters remained blinded to the genotypes during the experimental phase and the analysis. Adult male mice were group-housed in individually ventilated cages cages with food and water ad libitum in a regular 12-h light/dark cycle. Mice were weighed and inspected daily for general health. All behavioral experiments except for ErasmusLadder, Rotarod, LocoMouse paradigm, Y-maze, and eyeblink conditioning were performed in a behavioral box, a 130 × 80 × 80-cm box lined with 6-mm high-pressure laminate and soundproof foam. Experiments were recorded with a fixed camera (acA 1300-600gm; Basler AG, installed above the arenas) using the open-source software Bonsai (https://bonsai-rx.org). Experiments within the box were conducted under standard lighting, except for the observation of grooming behavior, which was conducted under infrared illumination. Unless stated otherwise, video recordings were analyzed with the open-source software OptiMouse (Ben-Shaul, 2017). Behavioral tasks were performed in the following order according to previously published protocols: (1) ErasmusLadder (Noldus; Vinueza Veloz et al., 2015). (2) Rotarod (Deacon, 2013); on days 1–4, the speed was accelerated to 40 revolutions per min and, on day 5, the speed was increased to 80 revolutions per min. (3) Three-chamber sociability and social novelty test (Yang et al., 2011); explorations were defined as periods when the experimental mouse was sniffing the stranger mouse and were scored by two independent, blinded observers, using the Observer XT (Noldus). (4) Self-grooming behavior (Badura et al., 2018); the duration and frequency of grooming bouts were manually scored by a blinded observer. Short bouts were defined as <3.5 s and long bouts as >3.5 s. (5) Open field test (Badura et al., 2018). (6) Elevated-plus maze (Badura et al., 2018). (7) Marble burying test (Wahl et al., 2022). (8) LocoMouse (Machado et al., 2015). (9) Y-maze (Badura et al., 2018); correct and incorrect choices were manually scored for each trial. A trial was considered correct if the mouse reached the hidden platform upon the first turn from the bottom arm into the correct arm with the platform. (10) Eyeblink conditioning (Badura et al., 2018; Giovannucci et al., 2017). All behavioral protocols are available upon request. Equipment was cleaned with 70% ethanol before testing the next animal. Cartoons displaying individual tasks were created with BioRender.com.

### LocoMouse analysis

Data collection was performed in Pylon viewer (Basler). DeepLabCut (DLC; https://github.com/DeepLabCut/DeepLabCut) was used for tracking individual body parts and the analysis of locomotion dynamics. The front and hind paws as well as the nose and tail base were used for tracking body movements. In total, 20 frames of 10 different videos from different mice walking in both directions were extracted (a total of 200 frames). Frames were manually labeled with the aforementioned body parts. These frames were used for training in the pretrained deep neural network ResNet50 (He et al., 2016; Insafutdinov et al., 2016). Evaluation of the network was done to confirm a low error in pixels between labeled frames and predictions. 15 videos per mouse and in each walking direction (left to right and right to left) were analyzed. DLC generates a matrix with x and y positions in pixels for each body part. This matrix was used to calculate movement parameters using a custom Python code (https://github.com/BaduraLab/DLC_analysis) based on previously published parameters (Machado et al., 2015).

### Acquisition and analysis of human and mouse MRI data

Human MRI data was collected at a resolution of ∼1 mm (0.9 × 0.85 × 0.85 mm) with a 7 T scanner (Achieva 7T, Philips) equipped with an Tx8/Rx32 rf-coil (Nova Medical). A T1-weighted MPRAGE sequence was used to obtain good gray-white matter contrast in minimal scan time. Three 2-min acquisitions were averaged after coregistration with SPM12 to boost signal strength and limit the scanning time to ensure patient comfort. Skull stripping was performed with SPM12. The human cerebellum was semiautomatically segmented with the SUIT toolbox (Diedrichsen, 2006). The annotation was subsequently manually checked and corrected, if necessary (Diedrichsen and Zotow, 2015). Human subcortical structures were annotated by linearly and nonlinearly transforming subject brains to the CIT168 atlas.

To acquire the mouse MRI data, the brains within the intact skull were processed by soaking them in PBS (pH 7.4) solution containing 1% Pro-Hance (Bracco Diagnostics) for 3 d at 4°C (or until transverse relaxation time [T_1_] of the brain tissue reached ∼150 ms). MRI was performed using a 15.2 T MR horizontal bore scanner and the BFG6S-100 actively shielded gradient system (1 T/m maximum gradient strength; Bruker BioSpin MRI). All scans were performed using 4-channel phase array coil for mouse head (Bruker, Biospin). Images were acquired using multi-echo spin echo sequences (repetition time/echo time = 350/26 ms, four averages) with field of view 18 × 14 × 8 mm with an imaging matrix 360 × 280 × 160, resulting in a spatial resolution of 50 × 50 × 50 mm. Skull stripping was performed semiautomatically with Rapid Automatic Tissue Segmentation and subsequent manual correction (Oguz et al., 2014). The mouse brain was annotated by linearly and nonlinearly transforming the mouse brains to an altered Allen atlas reference template (0.025 × 0.025 × 0.025 mm; Wang et al., 2020). The Allen atlas reference was altered by performing a linear transformation to the Australian Mouse Brain Mapping Consortium reference atlas (Janke and Ullmann, 2015). Cerebellar lobules and SN were manually corrected. One wild-type animal was excluded from comparison of non–manually adjusted structures because the scan showed high-intensity perfusion artifacts, which decreased the annotation accuracy of non–manually corrected structures.

Linear (affine) transformations were performed with FLIRT (FMRIB’s linear registration tool), and nonlinear transformations were performed with symmetric diffeomorphic registration (Avants et al., 2008; Jenkinson et al., 2012). Manual adjustment of MRI annotations was performed with ITK-SNAP (Yushkevich et al., 2006). Cerebellar adjustment for both human and mouse annotations was performed by first creating a cerebellar mask and using nearest neighbor interpolation to determine the lobule information, after which lobule annotation was additionally checked and annotated. Volumes were calculated in the native space of the original MRI scans for both human and mouse data.

### Histological analysis

Histological analysis was performed as previously described (Badura et al., 2013). All secondary antibodies were ordered from Jackson ImmunoResearch Europe. Pax5 staining was enhanced with the VectaFluor Excel Amplified Anti-Rabbit IgG DyLight 594 Antibody Kit (Vector Laboratories) according to the manufacturer’s instructions. Images were acquired with either an Axio Imager M2 (Carl Zeiss Microscopy) or an LSM 700 (Carl Zeiss Microscopy) confocal laser scanning microscope.

### smRNA FISH

Mice were anesthetized with pentobarbital and perfused with 0.9% NaCl followed by 4% paraformaldehyde. Brains were dissected from the skull and processed as described in the protocol of the manufacturer (Advanced Cell Diagnostics) with the following probes: Mm-Gad1-C3 (400951-C3), Mm-Gad2-C3 (439371-C3), Mm-Pax5-C2 (541761-C2), Mm-Rbfox3-C1 (313311-C1), and mCherry-O3-C2 (513021-C2). Images were acquired with either an Axio Imager M2 (Carl Zeiss Microscopy) for *Gad1&Gad2* single-stainings or an LSM 700 confocal laser scanning microscope (Carl Zeiss Microscopy) to determine the colocalization of the aforementioned markers.

### Automated cell quantification

Fluorescent microscopy images were processed and analyzed using Fiji (Schindelin et al., 2012) and SHARP-Track (Shamash et al., 2018 *Preprint*; https://github.com/BaduraLab/cell-counting). Segmentation was performed on the registered slices in Fiji using routine autothresholding methods. Afterwards, automated cell counting of positive neurons was performed to acquire the x and y coordinates of every detected cell. This output matrix was used to create a region of interest (ROI) array (1 cell = 1 ROI) per slice in SHARP-Track. This step allowed one-to-one matching between the ROI array and the previously registered slice. The reference-space locations and brain regions of each cell were obtained by overlapping the registration array with the ROI array. Cell counts were normalized by brain region area following the hierarchical structure of the Allen Brain Atlas.

### Statistical analysis

Statistical analysis was performed with GraphPad Prism 9 v9.2.0 or Python. All data were assessed for normal or log-normal distribution before choosing the appropriate test. An overview of all statistical tests, sample sizes, and P values is provided for all figures in the respective legends and Table S6. Human motor and MRI experiments were analyzed with a two-tailed one-sample *t* test in Python. The statistical evaluation of the RNA-seq data is described in Analysis of RNA-seq data.

### Online supplemental material

Fig. S1 shows the mutant DNA sequences inserted in the *Pax5*^R31Q^ and *Pax5*^E242^* alleles, the immunological characterization of *Pax5*^R31Q/E242^* and *Pax5*^R31Q/−^ mice, and AKT signaling in human B cells. Fig. S2 contains bioinformatic data explaining how the Pax5-R31Q mutation leads to deregulated gene expression in *Pax5*^R31Q/−^ pro-B cells. Fig. S3 contains behavioral data documenting the motor control, motor learning, social, and cognitive impairments of the *Pax5*^R31Q/−^ mouse. Fig. S4 contains the data of behavioral tests demonstrating the absence of behavioral abnormalities in B cell–deficient *Igh*^∆Jh/∆Jh^ mice. Fig. S5 describes the generation and characterization of the *Pax5*^Cre^ and *Rosa26*^HC^ alleles. Tables S1 and S2 contain the data of the immunological and neuropsychological assessments of the patient, respectively. Tables S3, S4, and S5 contain the RNA-seq data of all Pax5-regulated genes identified in *Pax5*^R31Q/+^, *Pax5*^+/−^, and *Pax5*^−/−^ pro-B cells, respectively, compared with *Pax5*^+/+^ pro-B cells. Table S6 contains the statistical data of all experiments of this study and the respective statistical methods used for their analyses. Table S7 contains oligonucleotide sequences used for PCR analysis and CRISPR-Cas9 mutagenesis. Table S8 describes all Illumina sequencing experiments generated for this study. Video 1 describes the visuomotor adaptation task used to generate the data of Fig. 6, A–D. Video 2 describes the visuomotor assessment task used to generate the data of Fig. 6, E and F. Video 3 describes the eyeblink conditioning test used to generate the data of Fig. 7, I and J.

## Supplementary Material

Table S1 shows flow-cytometric data of peripheral blood from the patient at the age of 11 yr.

Table S2 shows results of neuropsychological assessment and questionnaires (age of 19 yr).

Table S3 shows Pax5-activated genes that are no longer activated by Pax5-R31Q as shown by comparison of Pax5^+/+^ and Pax5^R31Q/−^ pro-B cells.

Table S4 shows Pax5-activated genes identified by RNA-seq comparison of Pax5^+/+^ and Pax5^+/−^ pro-B cells.

Table S5 shows Pax5-activated genes identified by comparison of Pax5^+/+^ and Pax5^−/−^ pro-B cells; Pax5^−/−^ = Vav-Cre Pax5^fl/fl^.

Table S6 shows statistical analysis of all experimental data.

Table S7 shows oligonucleotide sequence information.

Table S8 shows a description of all Illumina sequencing experiments generated for this study (GSE182463).

## Data Availability

The RNA-seq and ChIP-seq data generated for this study (Table S8) are available at GEO under accession no. GSE182463. The MRI data are deposited on GitHub (https://github.com/BaduraLab/MRI-Analysis). Custom-written code is also available on GitHub as indicated throughout Materials and methods.
