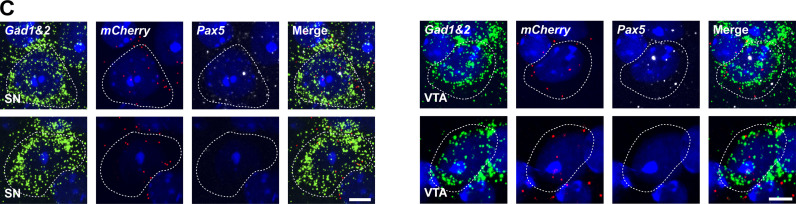# Correction: Biallelic *PAX5* mutations cause hypogammaglobulinemia, sensorimotor deficits, and autism spectrum disorder

**DOI:** 10.1084/jem.2022049812012022c

**Published:** 2022-12-07

**Authors:** Fabian M.P. Kaiser, Sarah Gruenbacher, Maria Roa Oyaga, Enzo Nio, Markus Jaritz, Qiong Sun, Wietske van der Zwaag, Emanuel Kreidl, Lydia M. Zopf, Virgil A.S.H. Dalm, Johan Pel, Carolin Gaiser, Rick van der Vliet, Lucas Wahl, André Rietman, Louisa Hill, Ines Leca, Gertjan Driessen, Charlie Laffeber, Alice Brooks, Peter D. Katsikis, Joyce H.G. Lebbink, Kikuë Tachibana, Mirjam van der Burg, Chris I. De Zeeuw, Aleksandra Badura, Meinrad Busslinger

Vol. 219, No. 9 | https://doi.org/10.1084/jem.20220498 | August 10, 2022

The authors regret that in the original version of Fig. 10 C, the *Gad1&2*, *mCherry*, and *Pax5* smRNA-FISH data of the SN neuron had been accidentally copied from the bottom left row into the bottom right row. The corrected Fig. 10 C is shown here. The contours of the VTA neuron in the bottom right row remain unchanged, and the corrections do not alter the conclusions at the end of the Results section: “smRNA FISH analysis revealed concomitant expression of *mCherry* and *Gad1*, *Gad2* mRNA in the VTA and SN in cells with and without active Pax5 expression (Fig. 10 C), demonstrating that GABAergic neurons in these regions without active *Pax5* expression also originate from *Pax5*-expressing progenitor cells.” The errors appear in print and in PDFs downloaded before December 1, 2022.

**Figure. 10:**