# Supplementary material for: Biallelic PAX5 mutations cause hypogammaglobulinemia, sensorimotor deficits, and autism spectrum disorder
Source: J Exp Med. 2022 Aug 10;219(9):e20220498. doi: 10.1084/jem.20220498 (PMC9372349; doi:10.1084/jem.20220498)
Supplement: Table S1 — shows flow-cytometric data of peripheral blood from the patient at the age of 11 yr. [file JEM_20220498_TableS1.pdf]

Table S1: Flow-cytometric data of peripheral blood from the patient at the age of 11 years

Cell population	Absolute count (x 10^9)	Normale range	Percentage of parental population
WBC	7.8		
Lymphocytes	1.7	1.0-5.3	22.2% (within leucocyte gate)
• T lymphocytes ($CD3^+$)	1.73	0.8-3.5	88.3% of lymphocytes
• NK cells ($CD16^+$ $CD56^+$ $CD3^−$)	0.17	0.07-1.2	8.7% of lymphocytes
• B lymphocytes ($CD19^+$)	0.06	0.2-0.6	3.1% of lymphocytes
B-lymphocyte subsets (Within $CD19^+$ gate)			
Transitional (IgD^+ $CD27^−$ $CD24^+$ $CD38^+$)	14	4-108	23.8%
Naïve mature (IgD^+ $CD27^−$ $CD24^{dim}$ $CD38^{dim}$)	30	87-390	49.7%
Marginal zone/natural effector (IgD^+ $CD27^+$)	4	7-90	6.5%
Memory ($IgD^−$ $CD27^+$):	1	10-76	1.9%
• IgM	N/A	5-32%	5.7%
• IgG	N/A	25-74%	27.3%
• IgA	N/A	14-47%	67%
Plasmablasts			1%
T-lymphocyte subsets (Within $CD3^+$ gate)			
CD4+ T-lymphocytes	0.9	0.4-2.1	50.1%
• Naive ($CD45RO^−$ $CCR7^+$ $CD27^+$ $CD28^+$)			61.5%
• Central memory ($CD45RO^−$ $CCR7^+$ $CD27^+$ $CD28^+$)			11.8%
• Effector memory ($CCR7^−$)			26.2%
CD8+ T-lymphocytes	0.5	0.2-1.2	31.2%
• Naive ($CD45RO^−$ $CCR7^+$ $CD27^+$ $CD28^+$)			47.9%
• Central memory ($CD45RO^−$ $CCR7^+$ $CD27^+$ $CD28^+$)			1.2%
• Effector memory ($CCR7^−$)			51.2%
$HLA\text{-}DR^+$ T-lymphocytes	0.04		2.6%
$TCR\alpha\beta^+$ $CD4^−$ $CD8^−$			1.6%
$TCR\alpha\beta^+$			80.1%
$TCR\gamma\delta^+$			19.8%
