# Supplementary material for: Biallelic PAX5 mutations cause hypogammaglobulinemia, sensorimotor deficits, and autism spectrum disorder
Source: J Exp Med. 2022 Aug 10;219(9):e20220498. doi: 10.1084/jem.20220498 (PMC9372349; doi:10.1084/jem.20220498)
Supplement: Table S2 — shows results of neuropsychological assessment and questionnaires (age of 19 yr). [file JEM_20220498_TableS2.pdf]

Table 2: Results of neuropsychological assessment and questionnaires (age of 19 years)

		Classification		
	**Score**	**Normal**	**Subclinical**	**Clinical**
**Neuropsychological tests**				
**WAIS-IV-NL**	z-score* (M=0.0; SD = 1.0)			
Total IQ	-1.9		X	
Verbal comprehension	-1.8		X	
Perceptual organization	-1.5		X	
Working memory	-1.7		X	
Processing speed	-1.6		X	
**ADOS-2, module 4**	Raw score			
Total score	11			X
**Self-report questionnaires**				
**SCL-90-R**	Raw score			
Total score	162			X
**BRIEF Self rating scale**	z-score			
Total score	-0.3	X		
**DCD-Q**	Raw score			
Total score	27			X
YSR	z-score			
Internalizing problems	-1.1		X	
Externalizing problems	-0.9	X		
Total problems	-1.9			X
**Parental questionnaires**				
**BRIEF Parental rating scale**	z-score			
Total score	-1.2		X	
**CBCL 6-18**	z-score			
Internalizing problems	-1.6			X
Externalizing problems	-1.0		X	
Total problems	-2.0			X
**SRS-2**	z-score			
	-3.1			X

*Lower z-scores represent worse performance/more problems.

For description of the instruments used for neuropsychological assessment see patient description in Materials and methods.
